# Supplementary material for: Design, synthesis and biological evaluation of N-oxide derivatives with potent in vivo antileishmanial activity
Source: PLoS One. 2021 Nov 1;16(11):e0259008. doi: 10.1371/journal.pone.0259008 (PMC8559926; doi:10.1371/journal.pone.0259008)
Supplement: S1 Appendix — (DOCX) [file pone.0259008.s001.docx]

Design, synthesis and biological evaluation of *N*-oxide derivatives with potent *in vivo* antileishmanial activity

Leandro da Costa Clementino^1,2^, Guilherme Felipe Santos Fernandes^1,2^, Igor Muccilo Prokopczyk^2^, Wilquer Castro Laurindo^1,2^, Danyelle Toyama^3^, Bruno Pereira Motta^2^, Amanda Martins Baviera^2^, Flávio Henrique-Silva^3^, Jean Leandro dos Santos^2*^, Marcia A S Graminha^2*^

^1^São Paulo State University (UNESP), Institute of Chemistry, Araraquara, Brazil.

^2^São Paulo State University (UNESP), School of Pharmaceutical Sciences, Araraquara, Brazil.

^3^Federal University of São Carlos, Department of Genetics and Evolution, São Carlos, Brazil.

Corresponding authors

* E-mail: jean.santos@unesp.br

* E-mail: marcia.graminha@unesp.br

**Appendix**

|  | **Figure** | **Page** |
| --- | --- | --- |
| ^1^H, ^13^C NMR Compound **4a** | Figs 1-2 | 2 |
| ^1^H, ^13^C NMR Compound **4b** | Figs 3-4 | 3 |
| ^1^H, ^13^C NMR Compound **4c** | Figs 5-6 | 4 |
| ^1^H, ^13^C NMR Compound **4d** | Figs 7-8 | 5 |
| ^1^H, ^13^C NMR Compound **4e** | Figs 9-10 | 6 |
| ^1^H, ^13^C NMR Compound **4f** | Figs 11-12 | 7 |
| ^1^H, ^13^C NMR Compound **4g** | Figs 13-14 | 8 |
| ^1^H, ^13^C NMR Compound **4h** | Figs 15-16 | 9 |
| ^1^H, ^13^C NMR Compound **4i** | Figs 17-18 | 10 |
| ^1^H, ^13^C NMR Compound **4j** | Figs 19-20 | 11 |
| ^1^H, ^13^C NMR Compound **4l** | Figs 21-22 | 12 |
| ^1^H, ^13^C NMR Compound **4m** | Figs 23-24 | 13 |
| ^1^H, ^13^C NMR Compound **4n** | Figs 25-26 | 14 |
| ^1^H, ^13^C NMR Compound **4o** | Figs 27-28 | 15 |
| ^1^H, ^13^C NMR Compound **14a** | Figs 29-30 | 16 |
| ^1^H, ^13^C NMR Compound **14b** | Figs 31-32 | 17 |
| ^1^H, ^13^C NMR Compound **14c** | Figs 33-34 | 18 |
| ^1^H, ^13^C NMR Compound **14d** | Figs 35-36 | 19 |
| ^1^H, ^13^C NMR Compound **14e** | Figs 37-38 | 20 |
| ^1^H, ^13^C NMR Compound **14f** | Figs 39-40 | 21 |
| ^1^H, ^13^C NMR Compound **14g** | Figs 41-42 | 22 |

**Fig 1.** ^1^H NMR spectrum (600 MHz, DMSO-d_6_) of compound **4a**


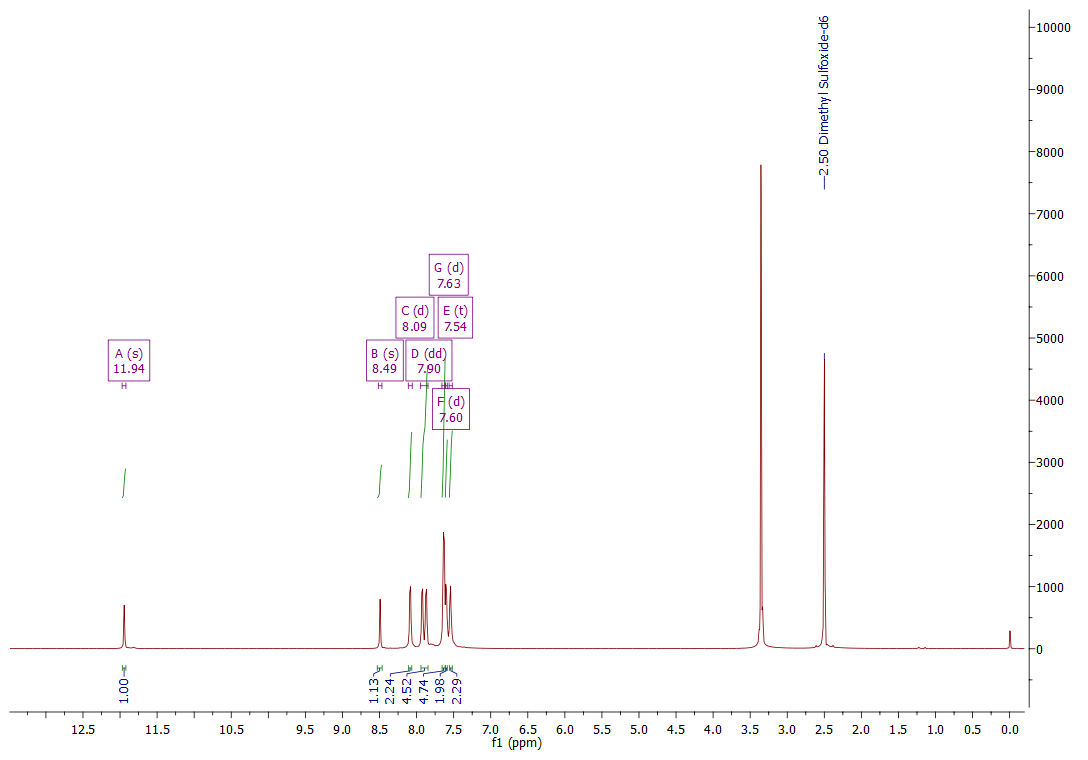


**Fig 2.** ^13^C NMR spectrum (151 MHz, DMSO-d6) of compound **4a**


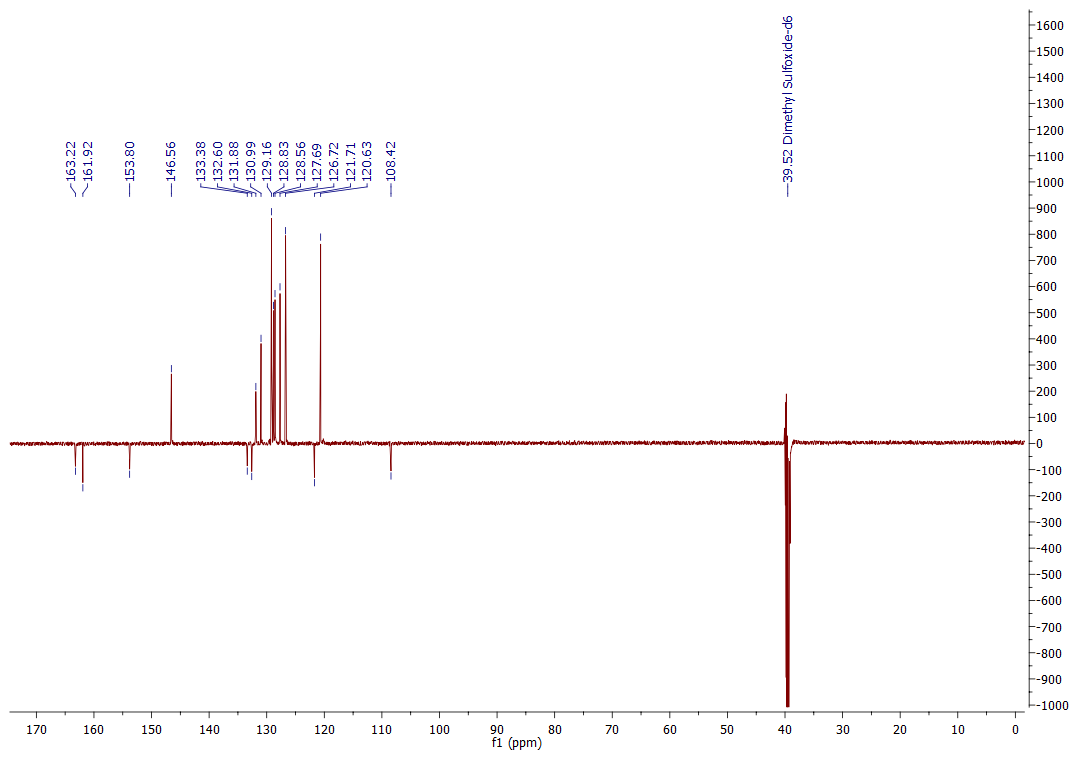


**Fig 3.** ^1^H NMR spectrum (600 MHz, DMSO-d6) of compound **4b**


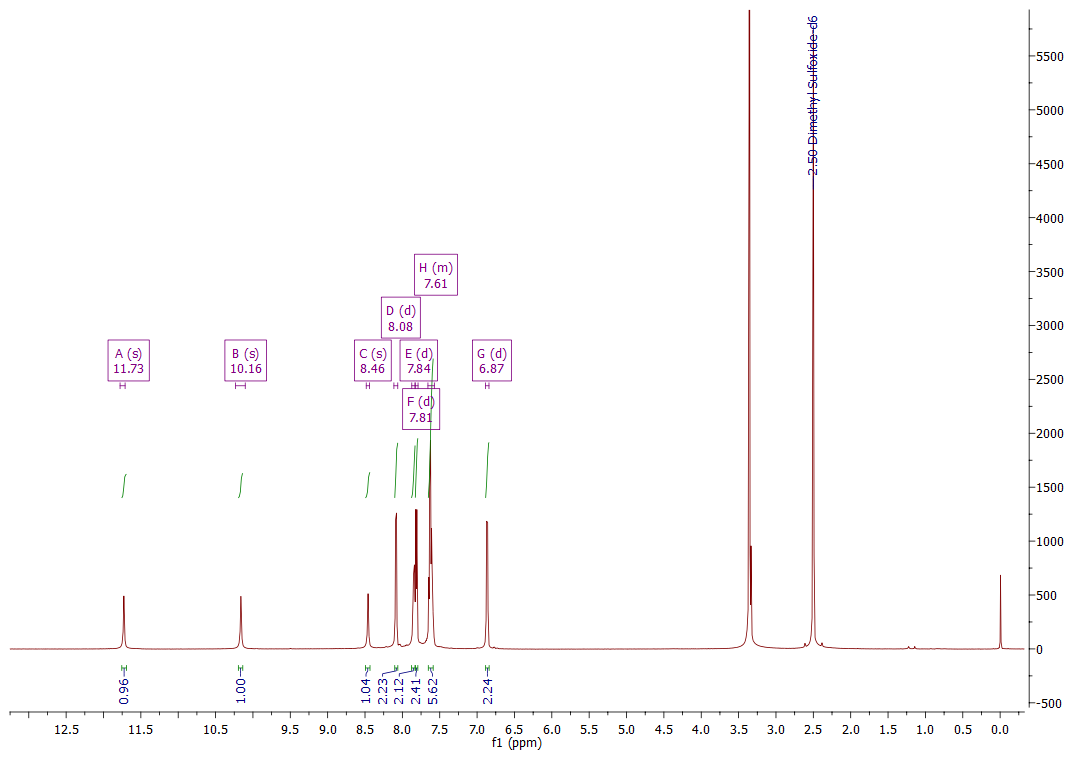


**Fig 4.** ^13^C NMR spectrum (151 MHz, DMSO-d6) of compound **4b**


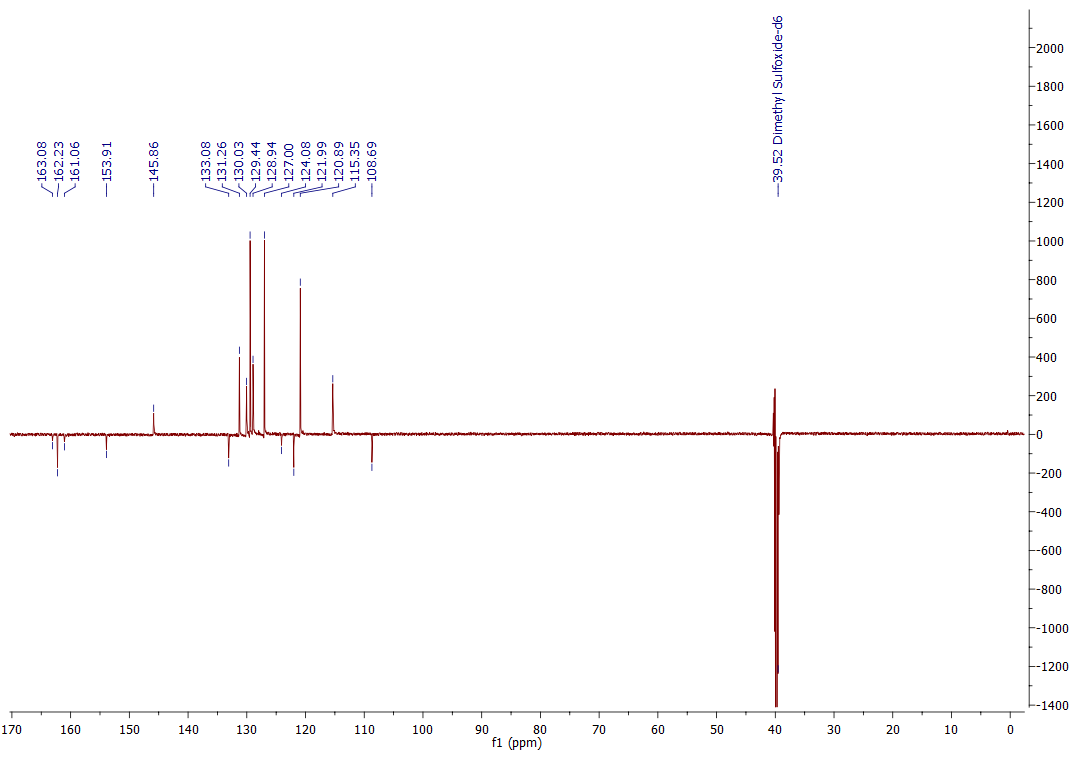


**Fig 5.** ^1^H NMR spectrum (600 MHz, DMSO-d6) of compound **4c**


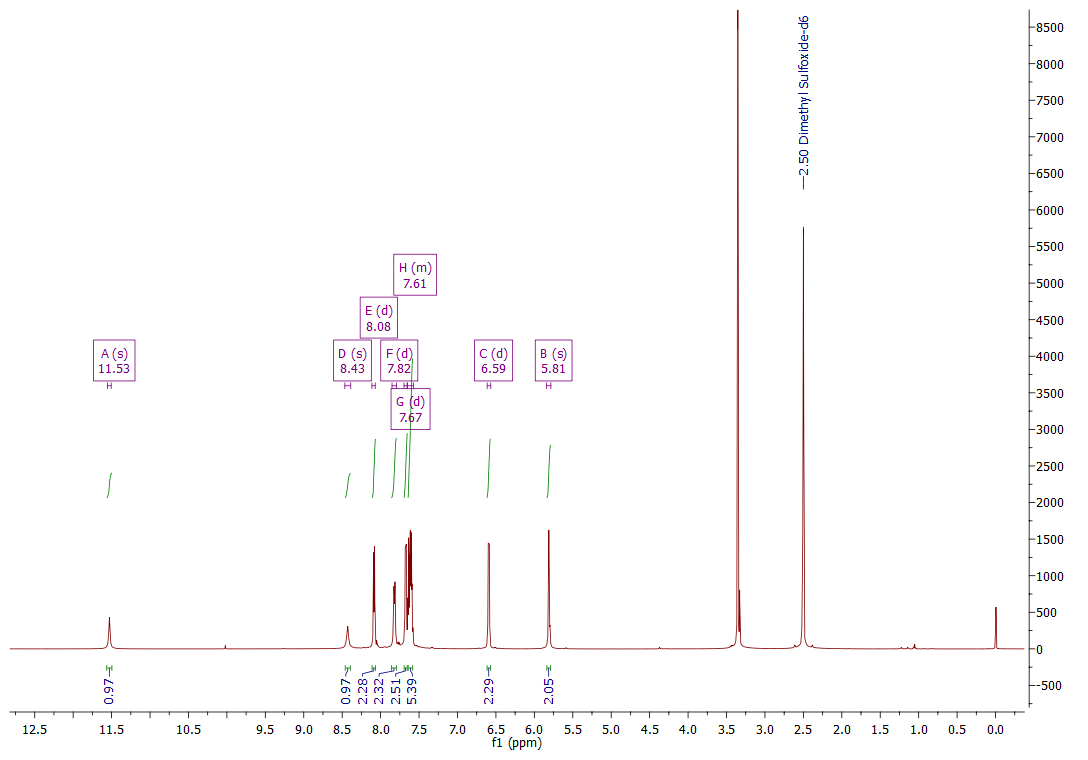


**Fig 6.** ^13^C NMR spectrum (151 MHz, DMSO-d6) of compound **4c**


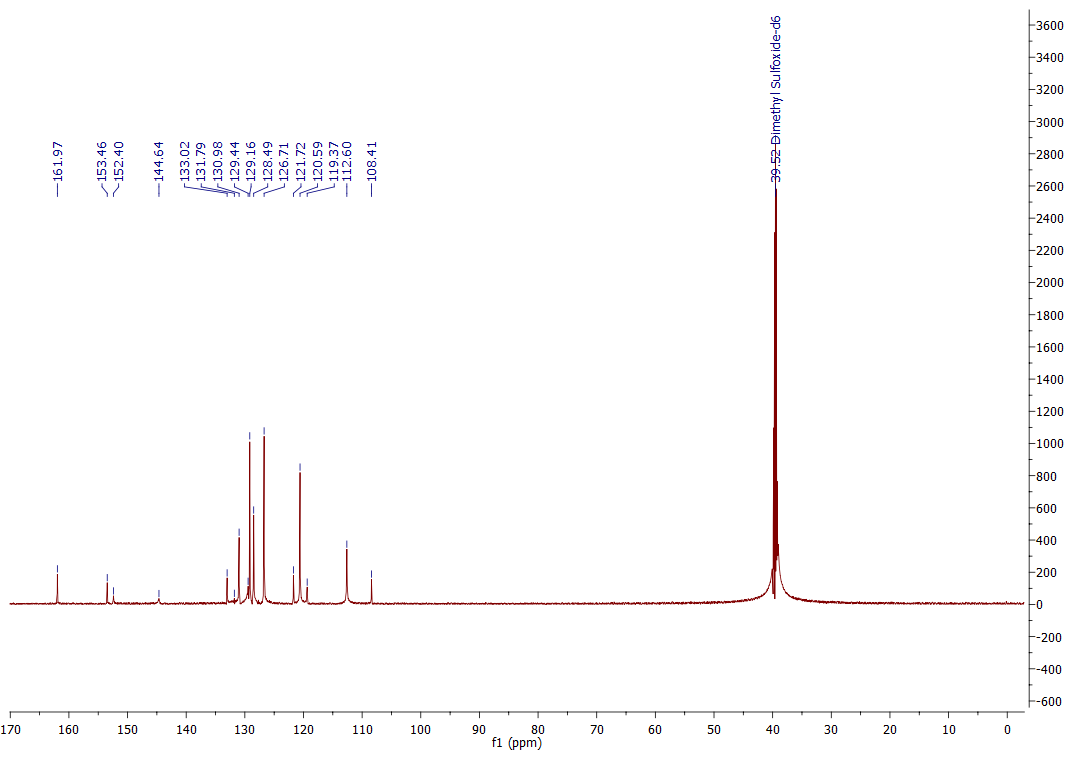


**Fig 7.** ^1^H NMR spectrum (600 MHz, DMSO-d6) of compound **4d**


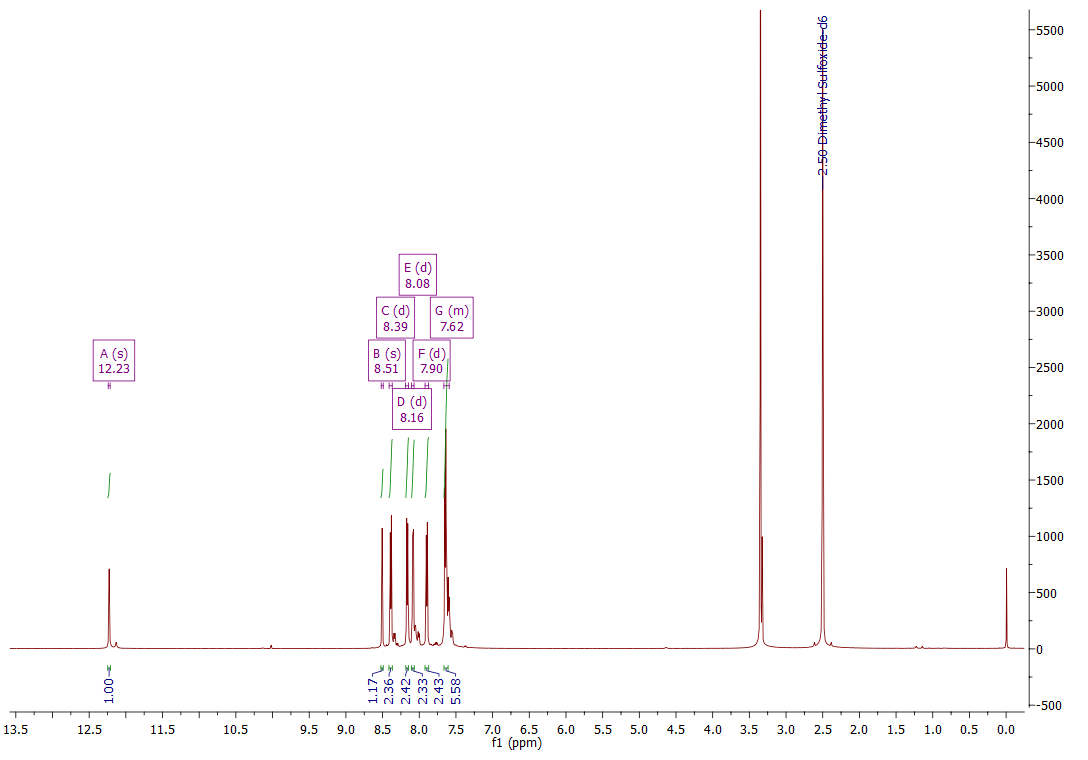


**Fig 8.** ^13^C NMR spectrum (151 MHz, DMSO-d6) of compound **4d**


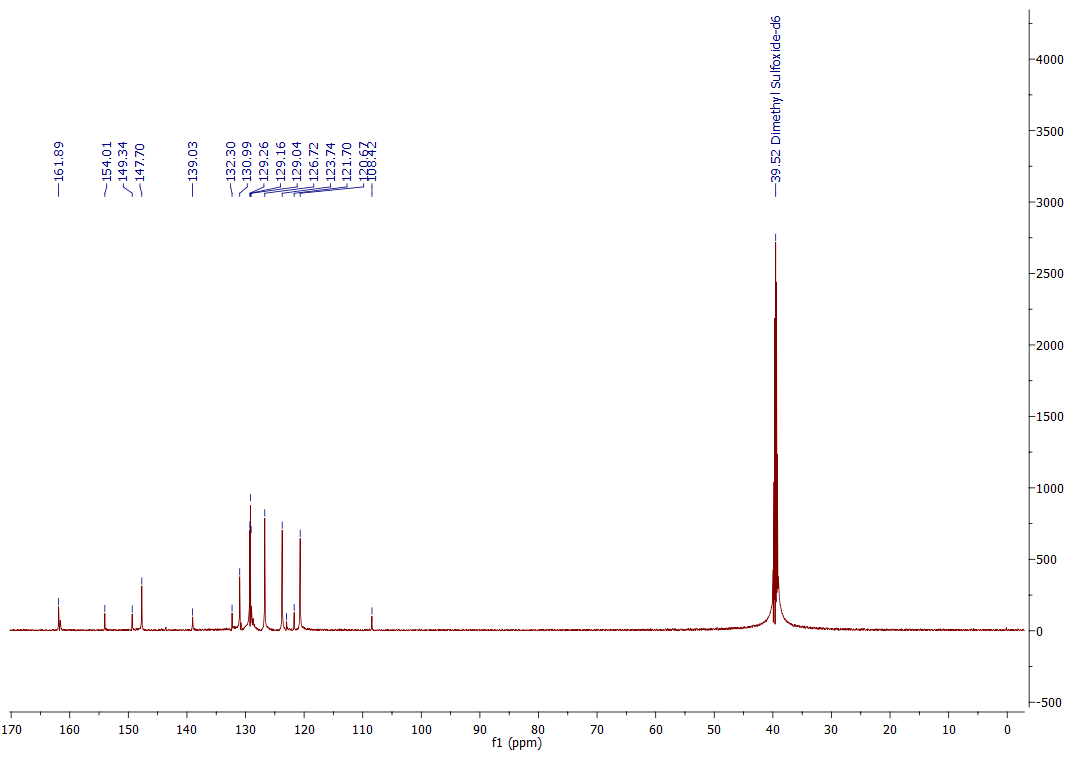


**Fig 9.** ^1^H NMR spectrum (600 MHz, DMSO-d6) of compound **4e**


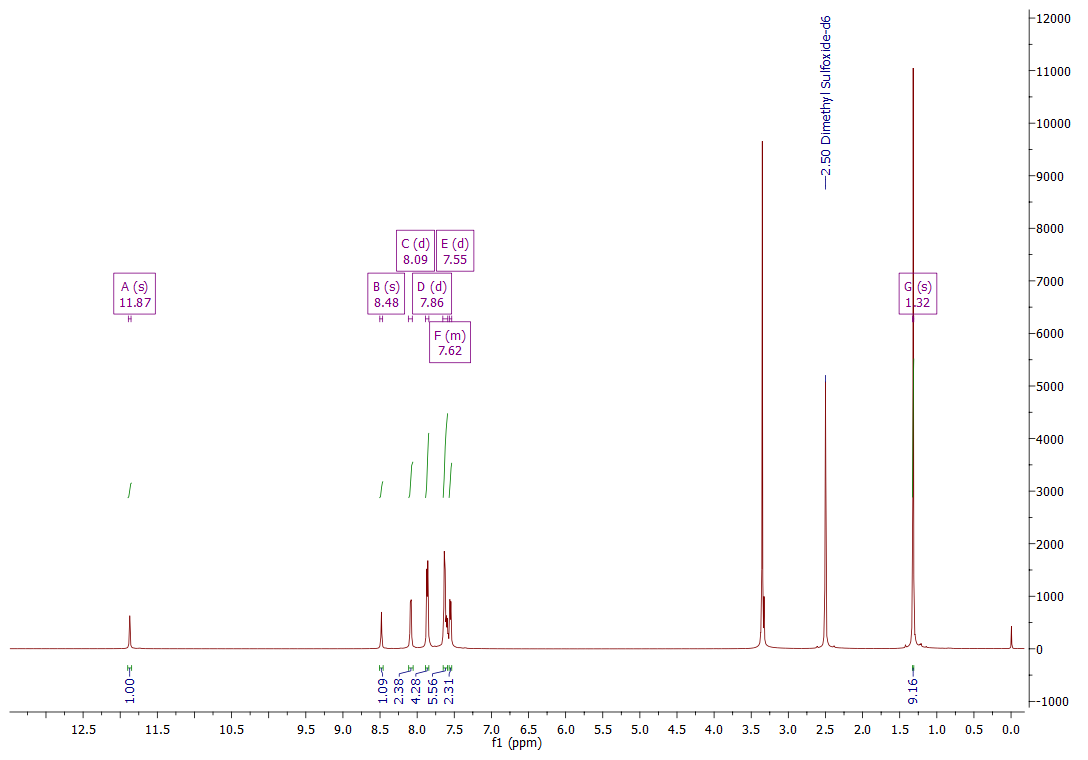


**Fig 10.** ^13^C NMR spectrum (151 MHz, DMSO-d6) of compound **4e**


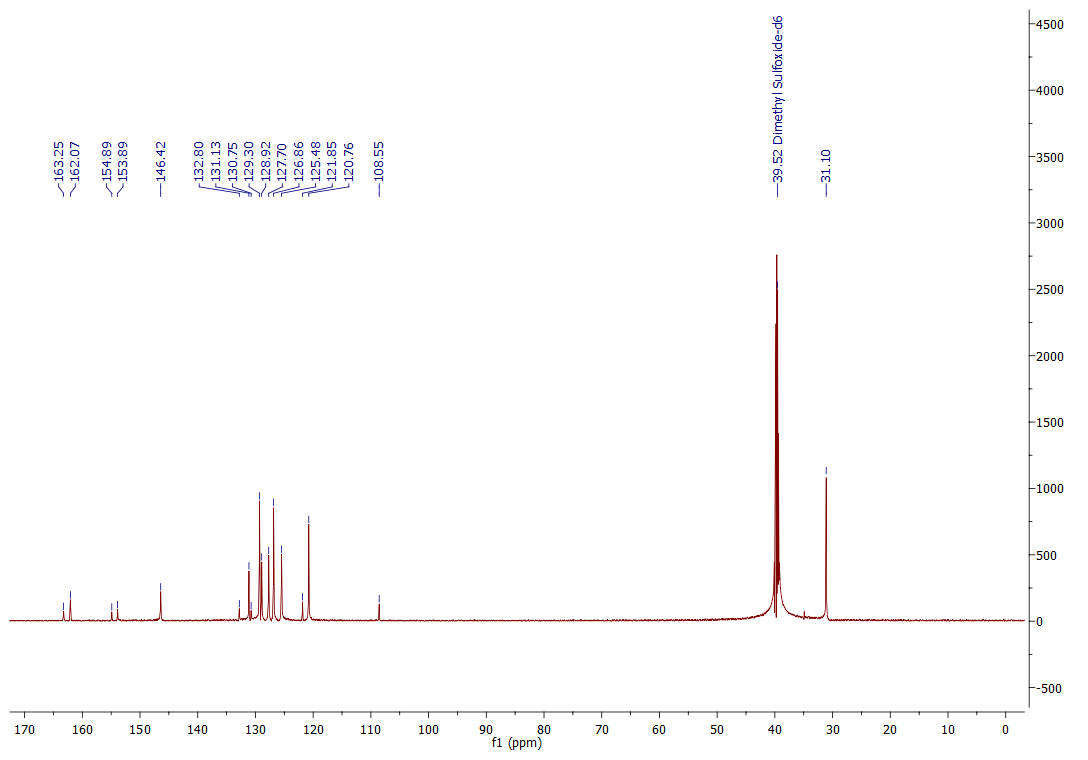


**Fig 11.** ^1^H NMR spectrum (600 MHz, DMSO-d6) of compound **4f**


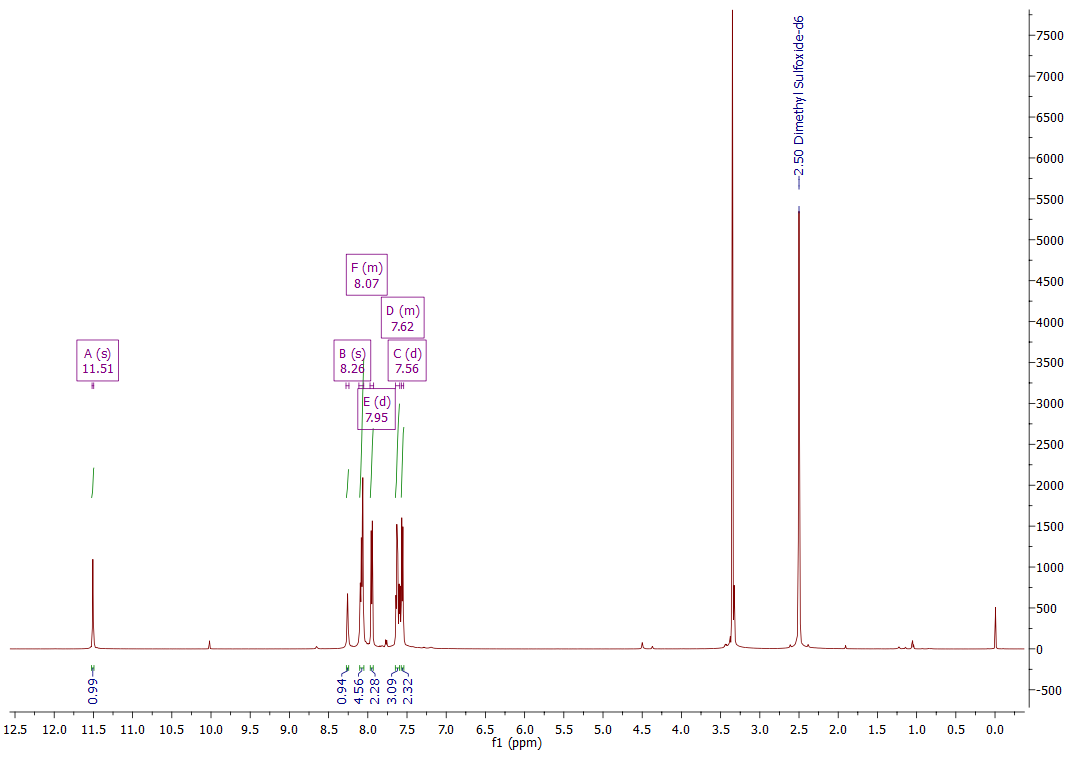


**Fig 12.** ^13^C NMR spectrum (151 MHz, DMSO-d6) of compound **4f**


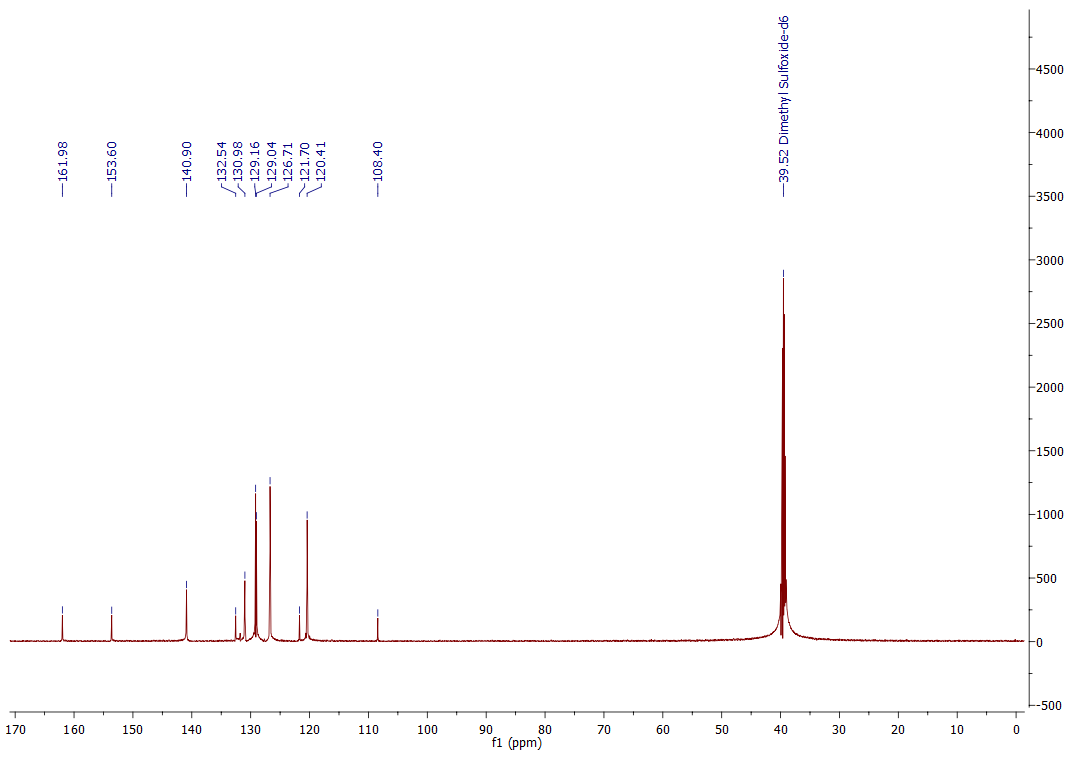


**Fig 13.** ^1^H NMR spectrum (600 MHz, DMSO-d6) of compound **4g**


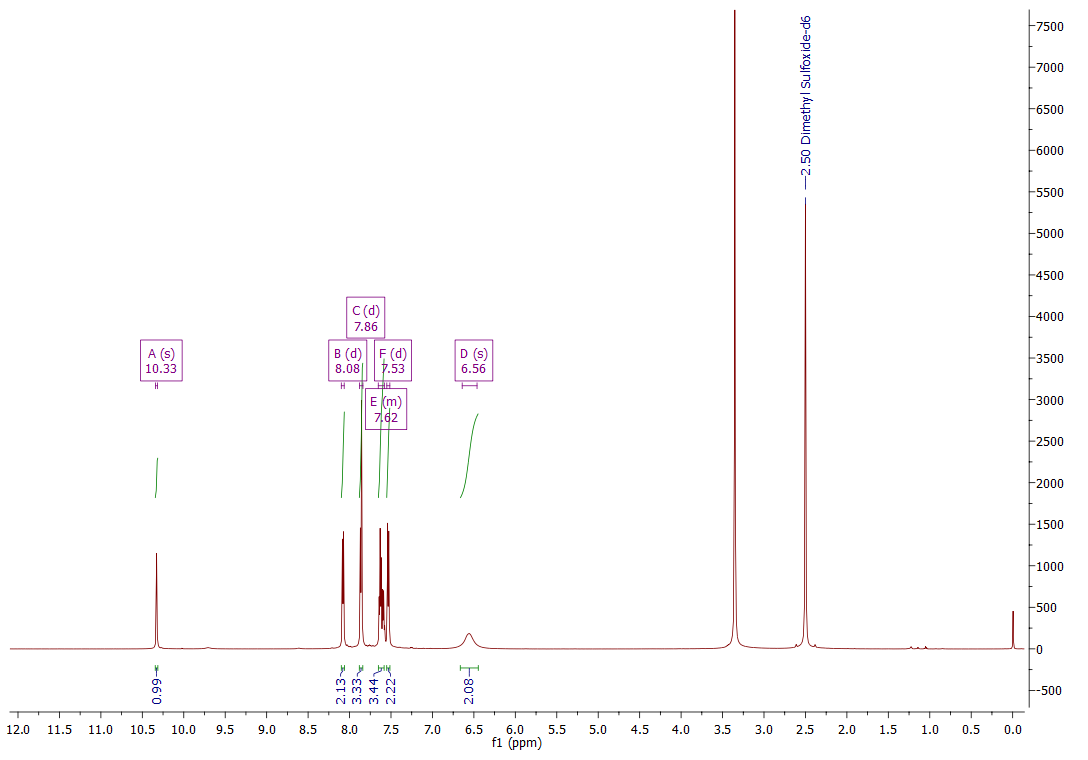


**Fig 14.** ^13^C NMR spectrum (151 MHz, DMSO-d6) of compound **4g**


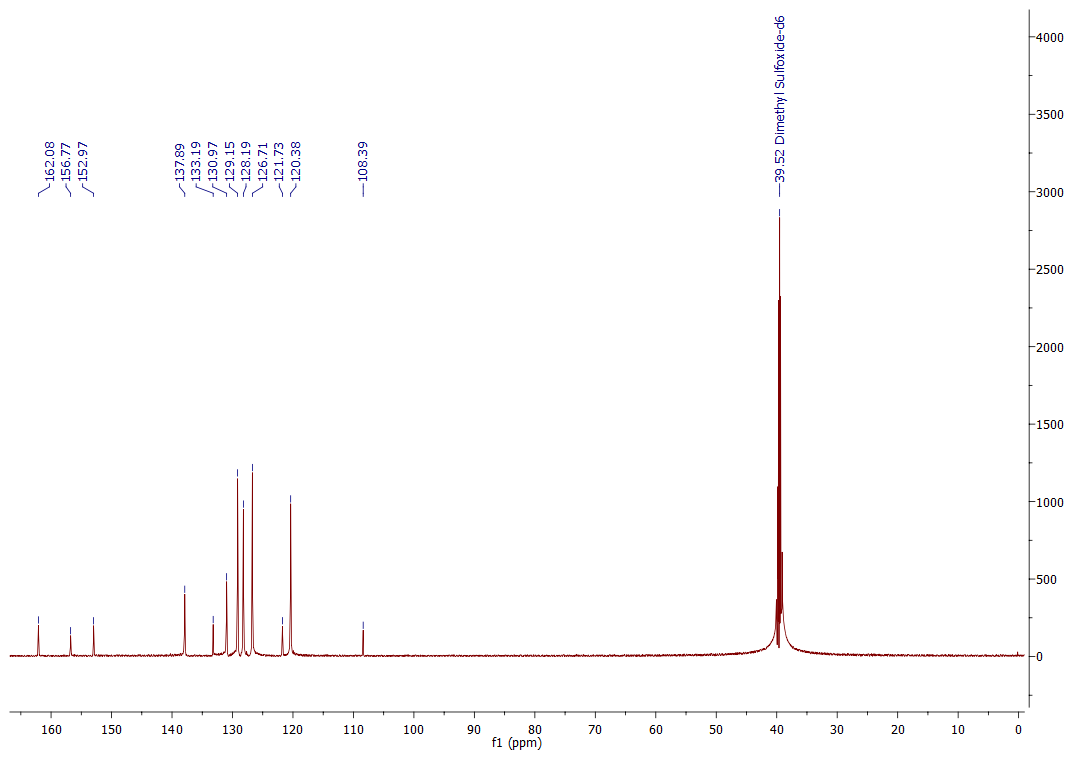


**Fig 15.** ^1^H NMR spectrum (600 MHz, DMSO-d6) of compound **4h**


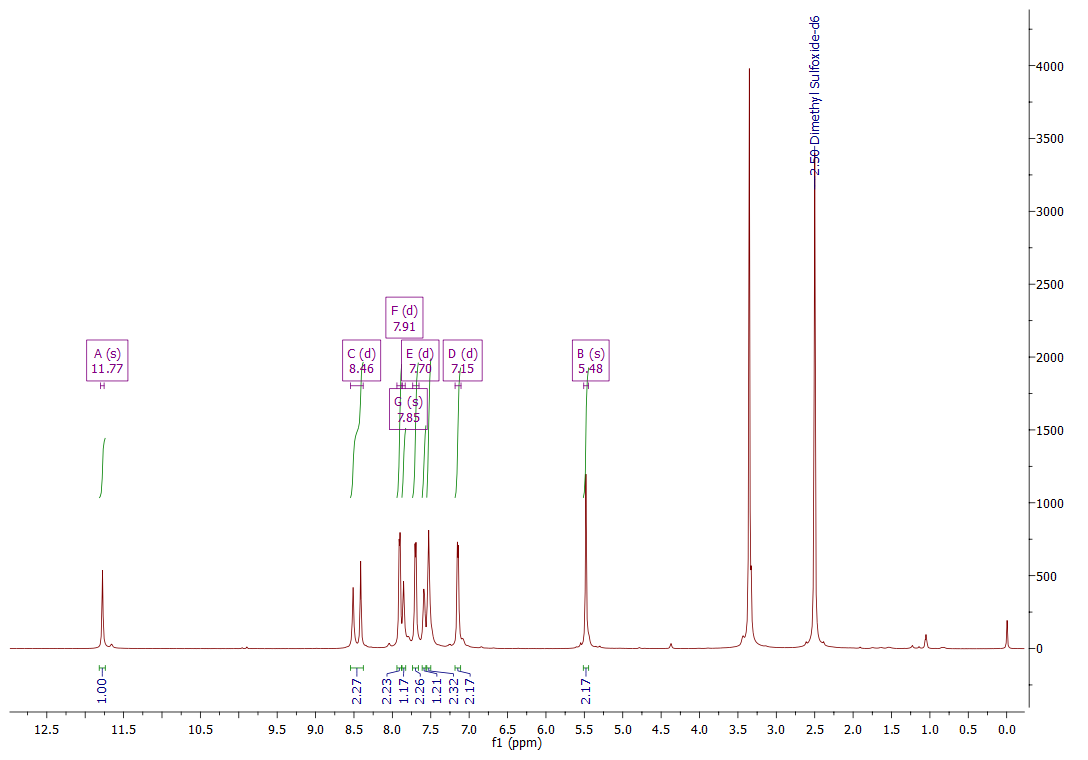


**Fig 16.** ^13^C NMR spectrum (151 MHz, DMSO-d6) of compound **4h**

**
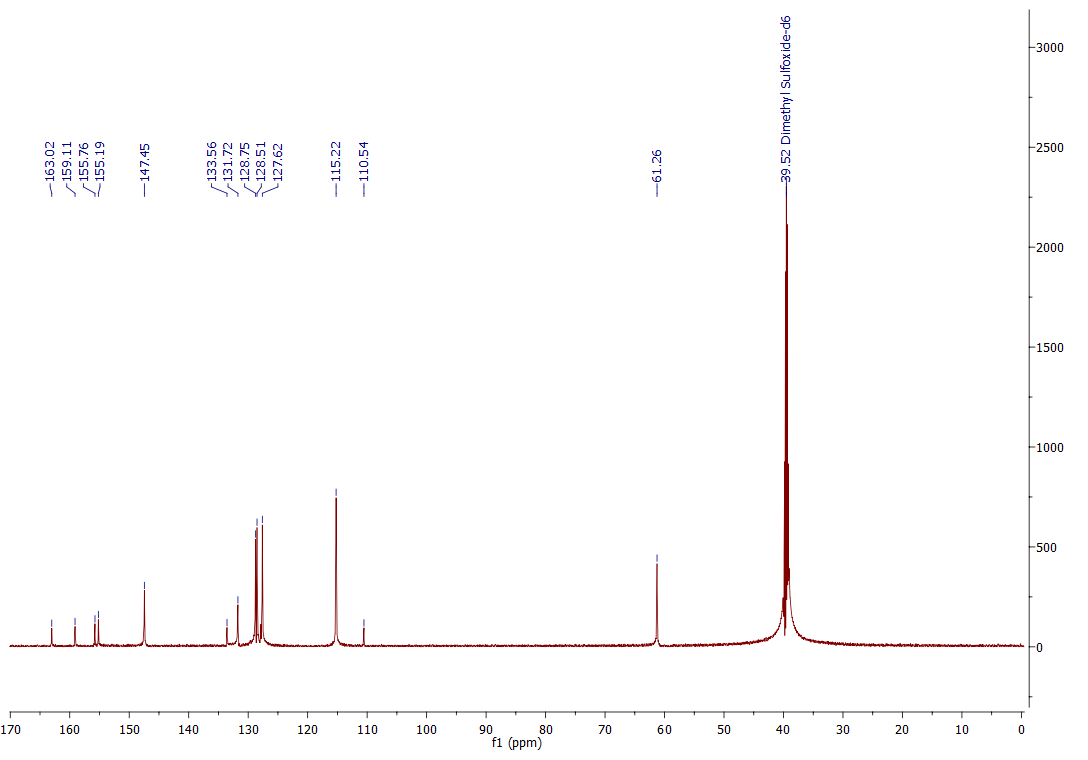
**

**Fig 17.** ^1^H NMR spectrum (600 MHz, DMSO-d6) of compound **4i**

**
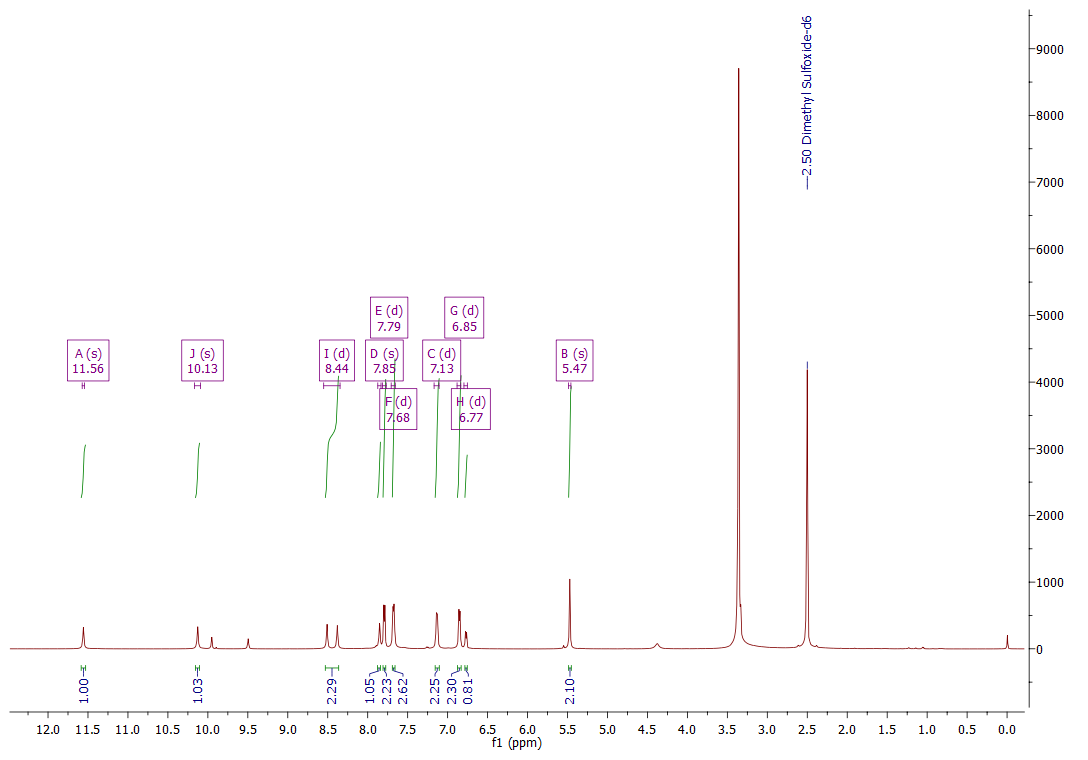
**

**Fig 18.** ^13^C NMR spectrum (151 MHz, DMSO-d6) of compound **4i**

**
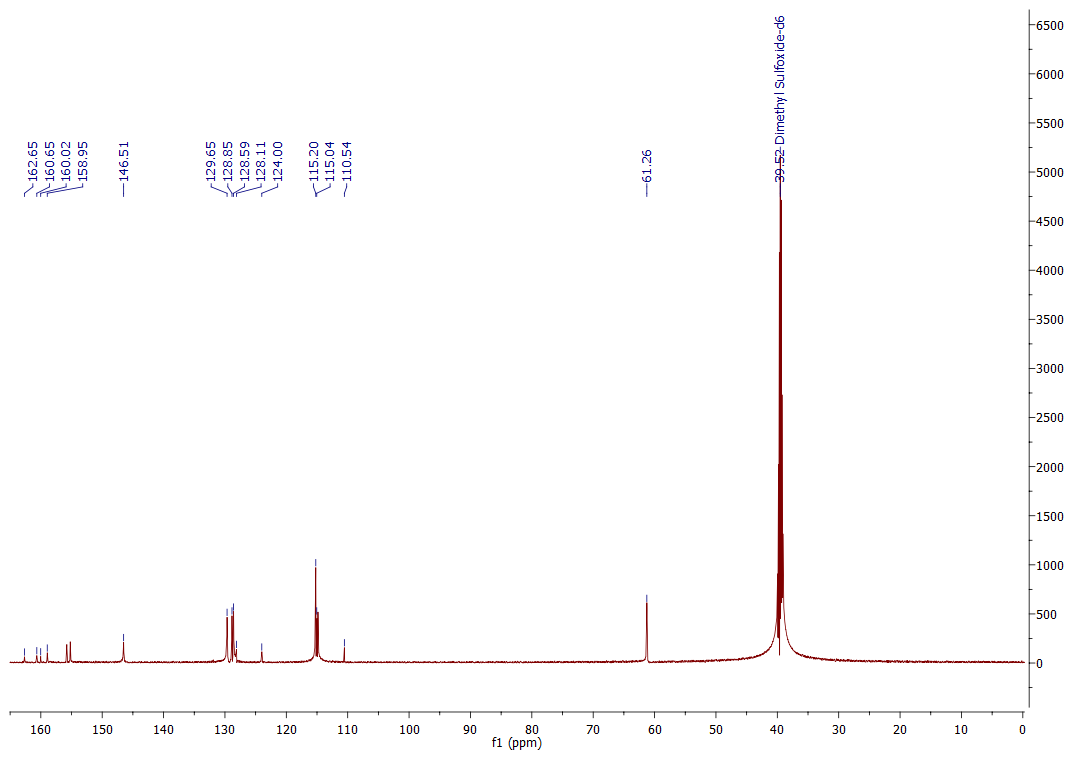
**

**Fig 19.** ^1^H NMR spectrum (600 MHz, DMSO-d6) of compound **4j**

**
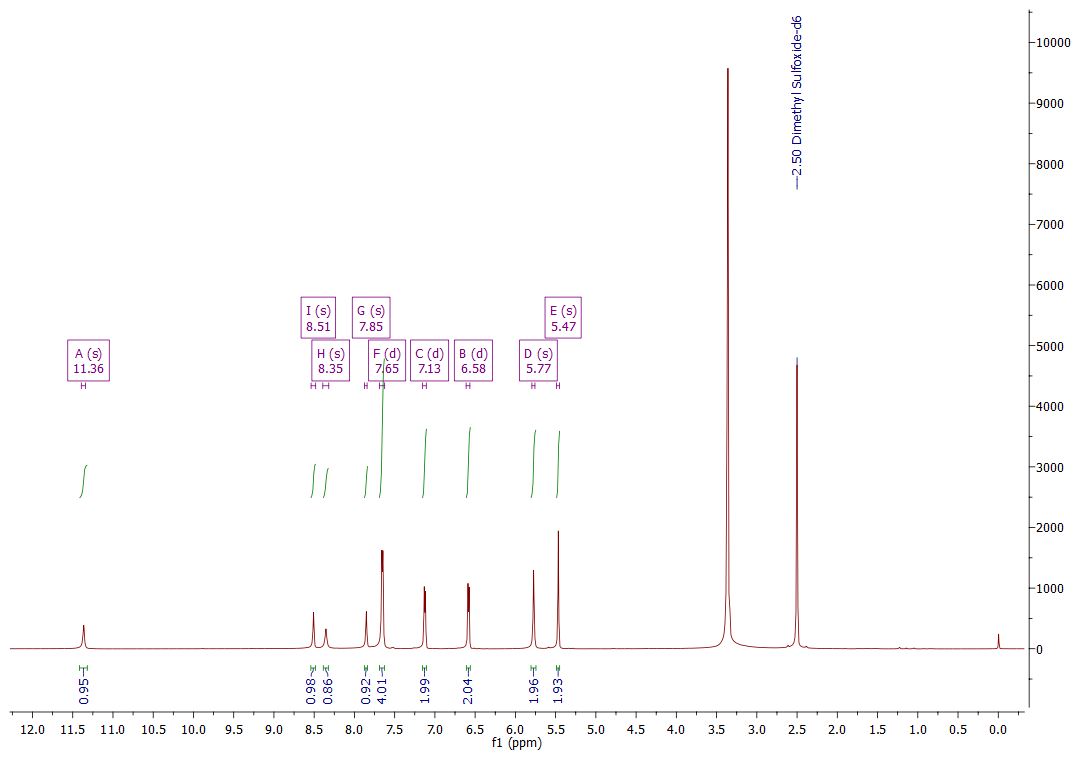
**

**Fig 20.** ^13^C NMR spectrum (151 MHz, DMSO-d6) of compound **4j**

**
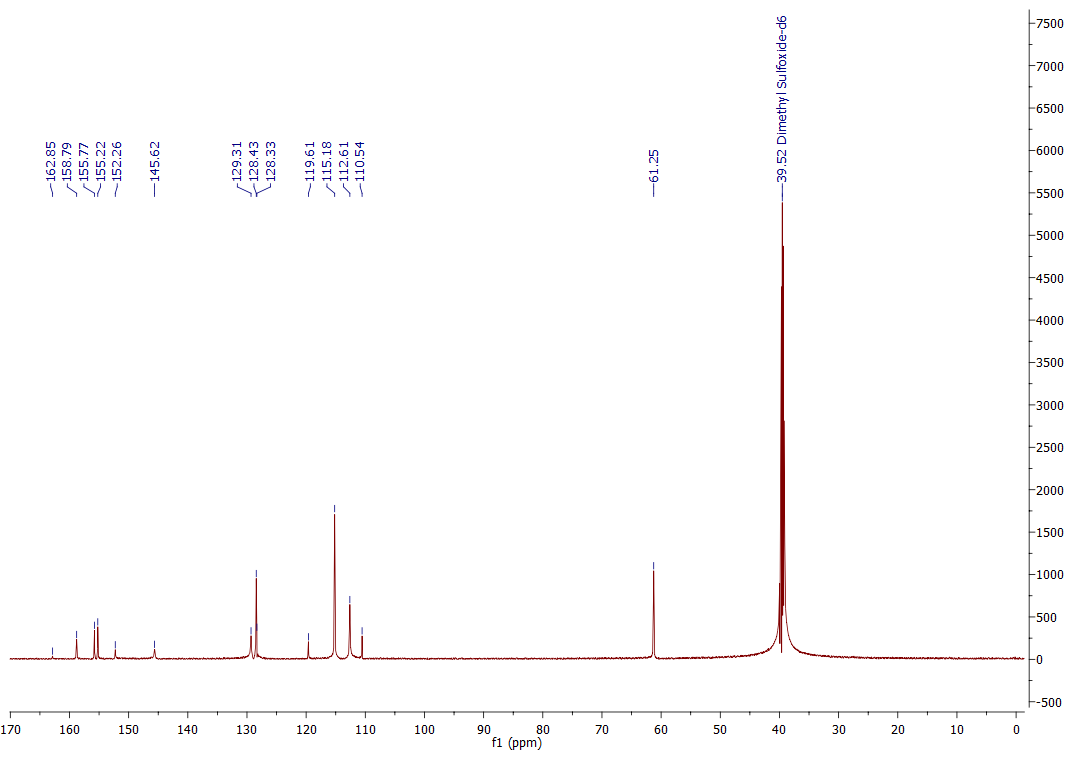
**

**Fig 21.** ^1^H NMR spectrum (600 MHz, DMSO-d6) of compound **4l**

**
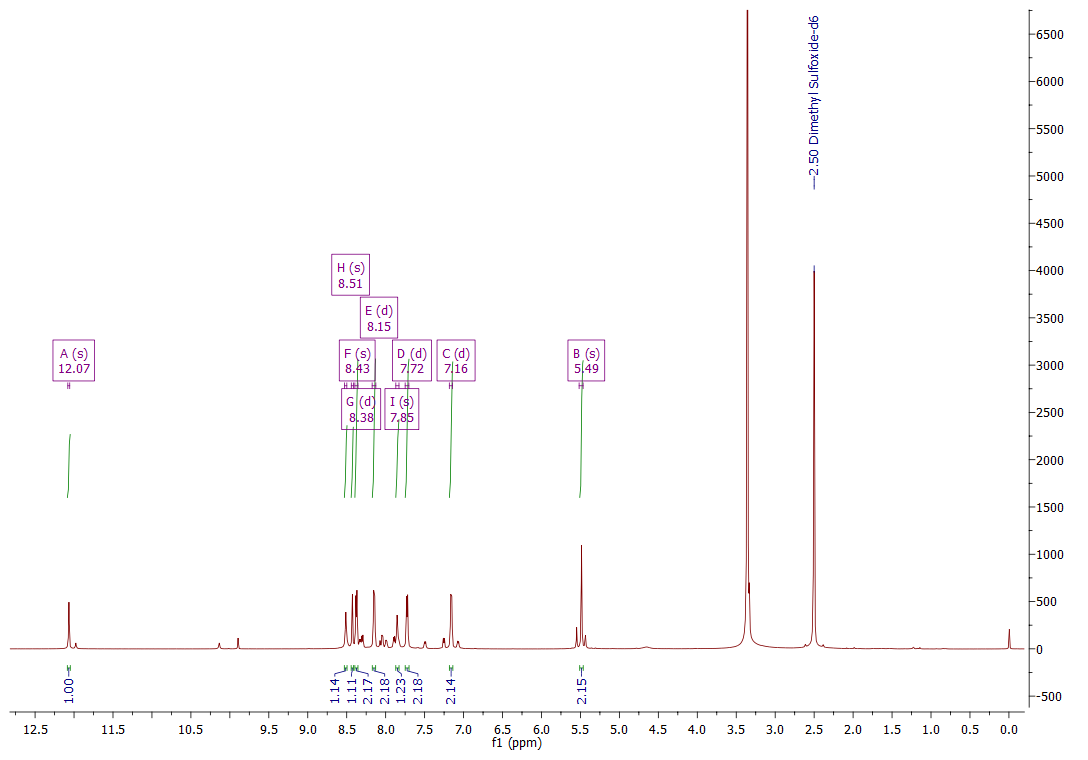
**

**Fig 22.** ^13^C NMR spectrum (151 MHz, DMSO-d6) of compound **4l**

**
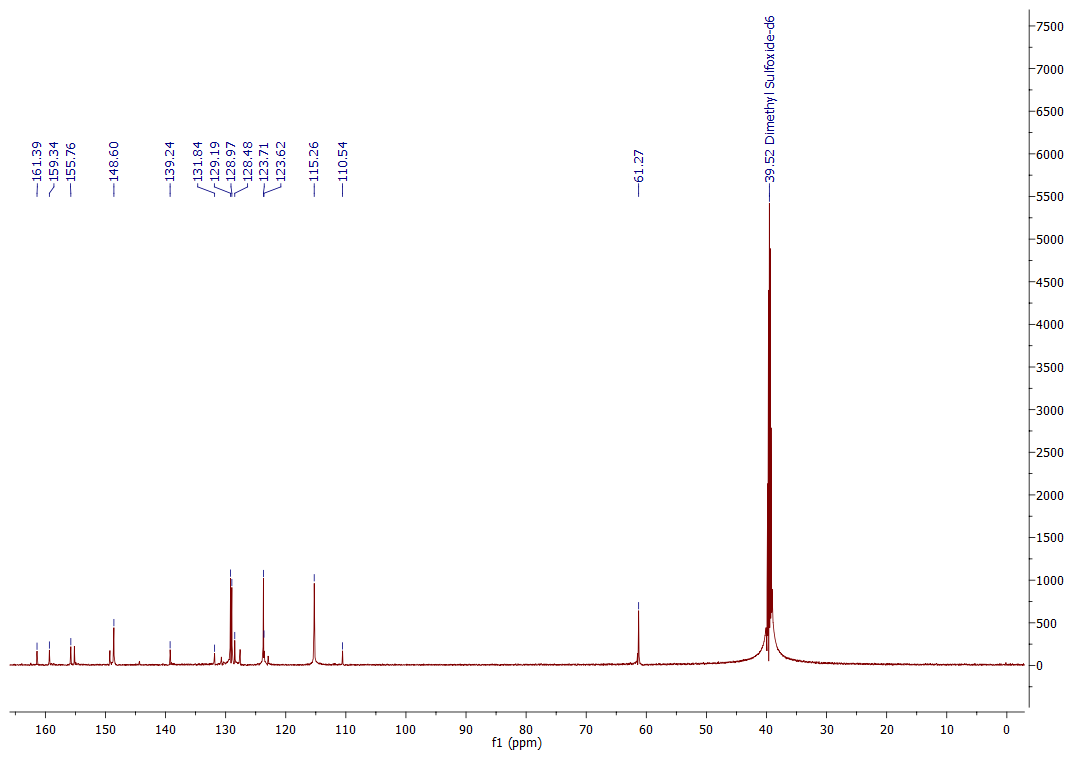
**

**Fig 23.** ^1^H NMR spectrum (600 MHz, DMSO-d6) of compound **4m**

**
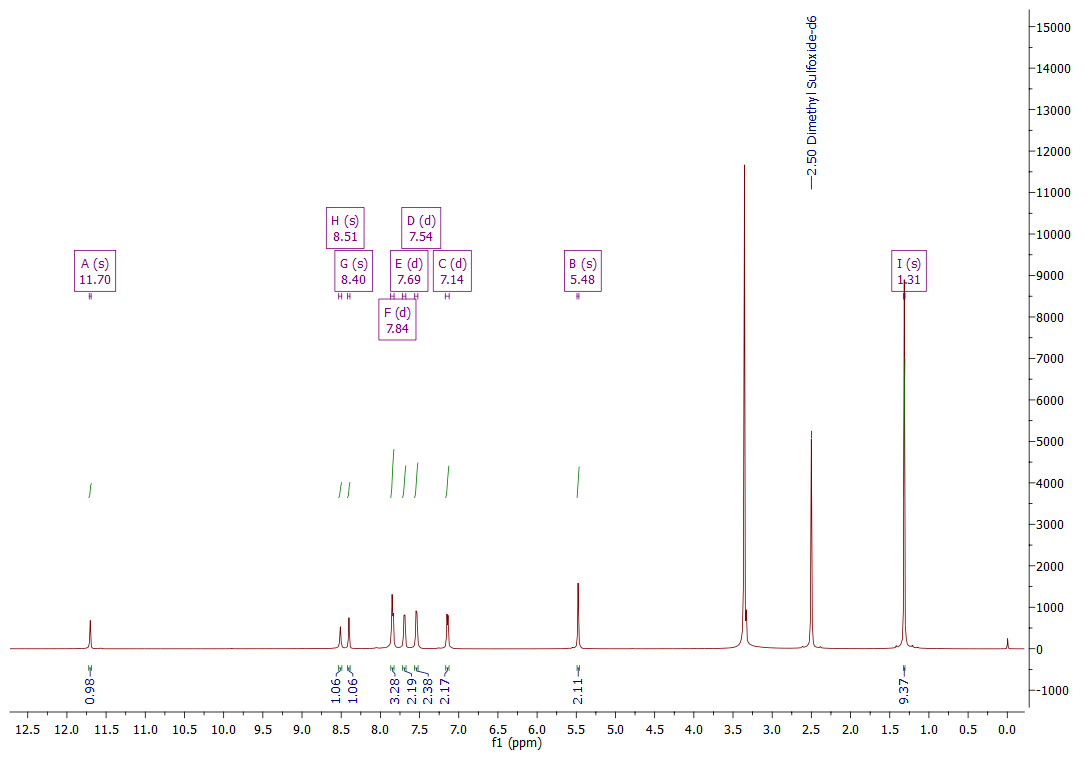
**

**Fig 24.** ^13^C NMR spectrum (151 MHz, DMSO-d6) of compound **4m**

**
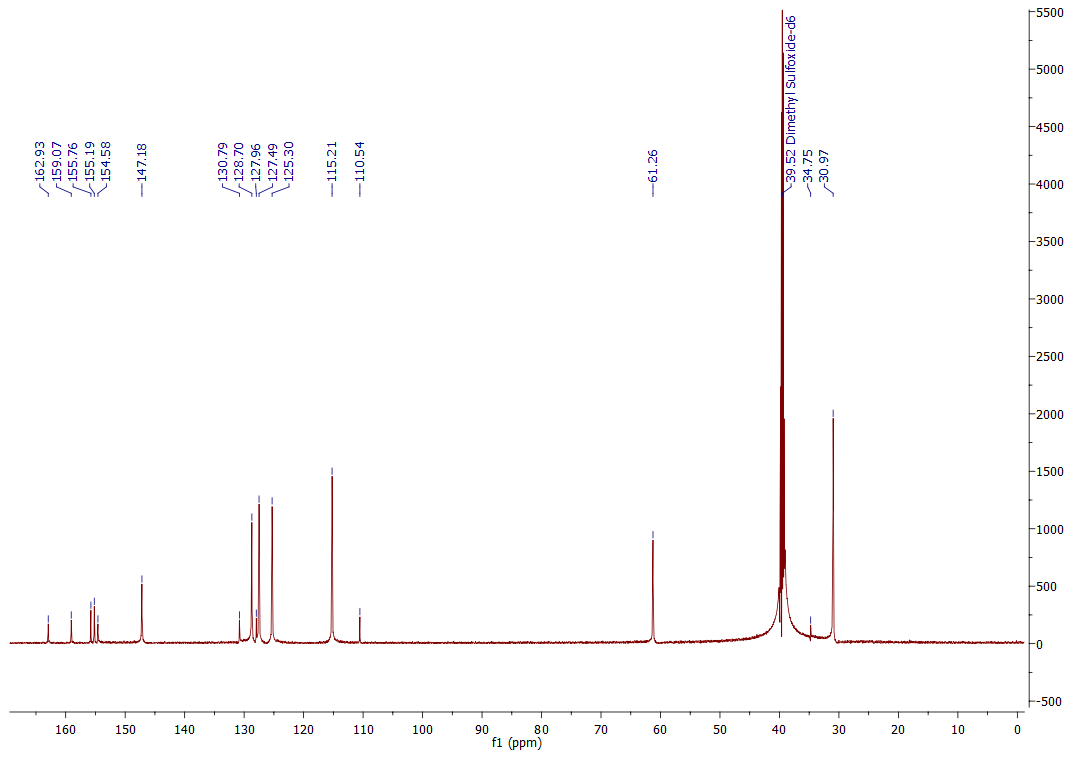
**

**Fig 25.** ^1^H NMR spectrum (600 MHz, DMSO-d6) of compound **4n**

**
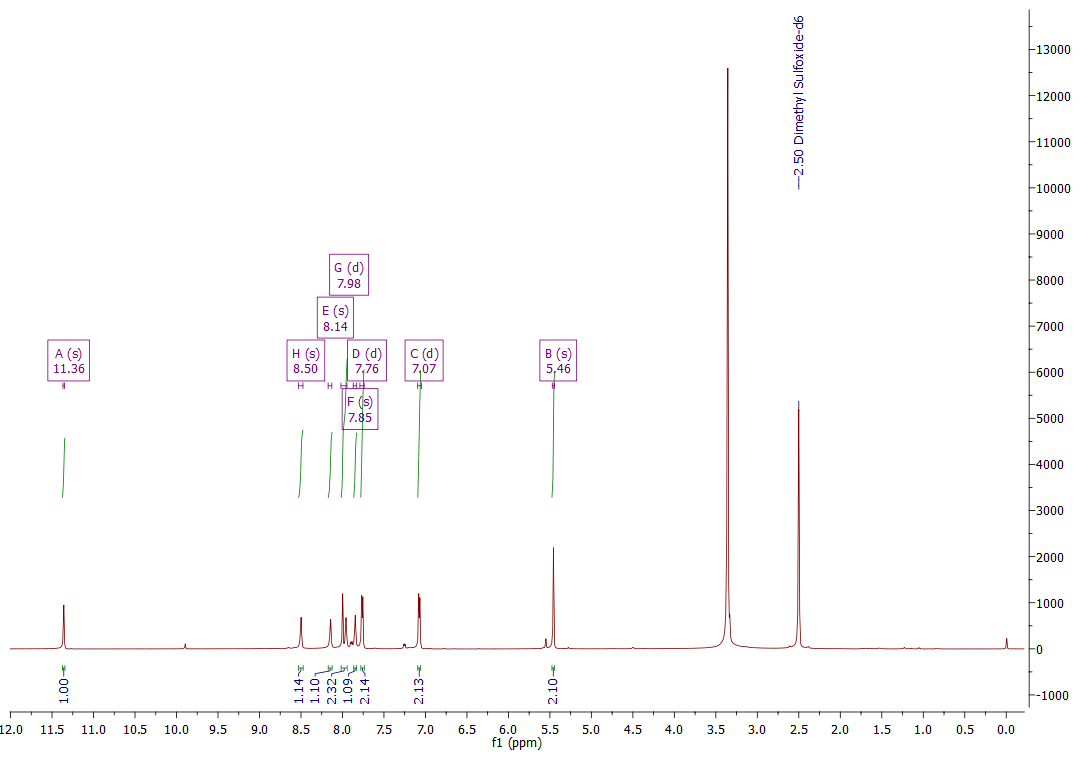
**

**Fig 26.** ^13^C NMR spectrum (151 MHz, DMSO-d6) of compound **4n**

**
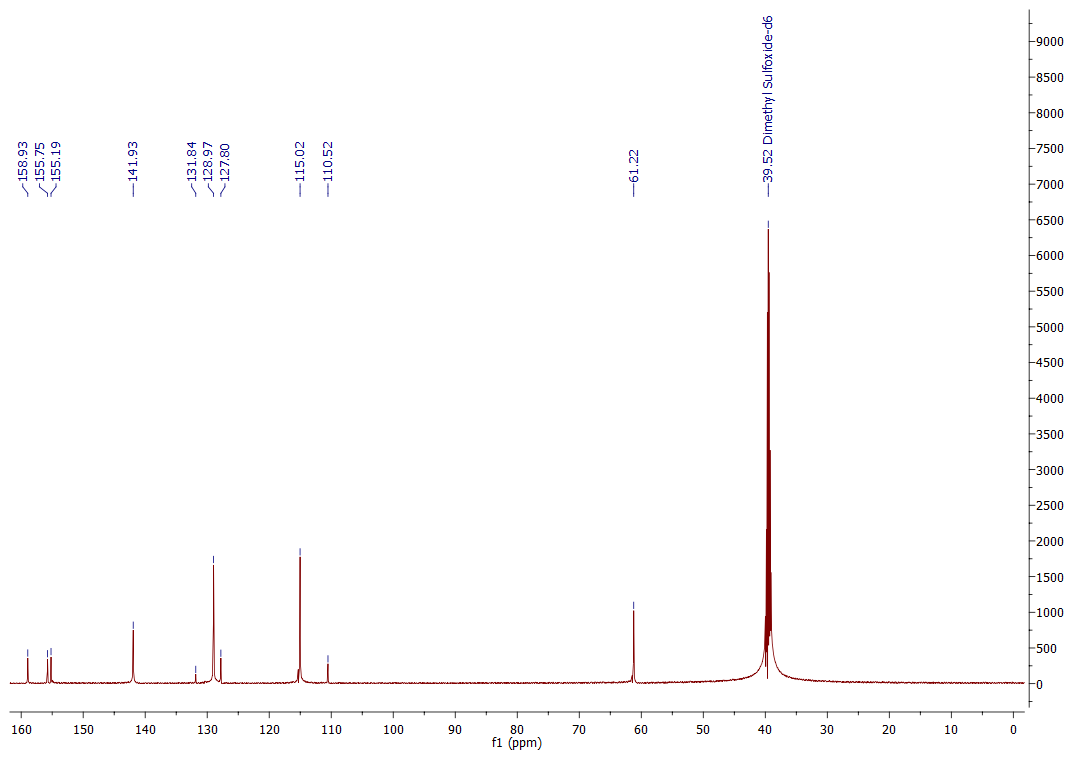
**

**Fig 27.** ^1^H NMR spectrum (600 MHz, DMSO-d6) of compound **4o**

**
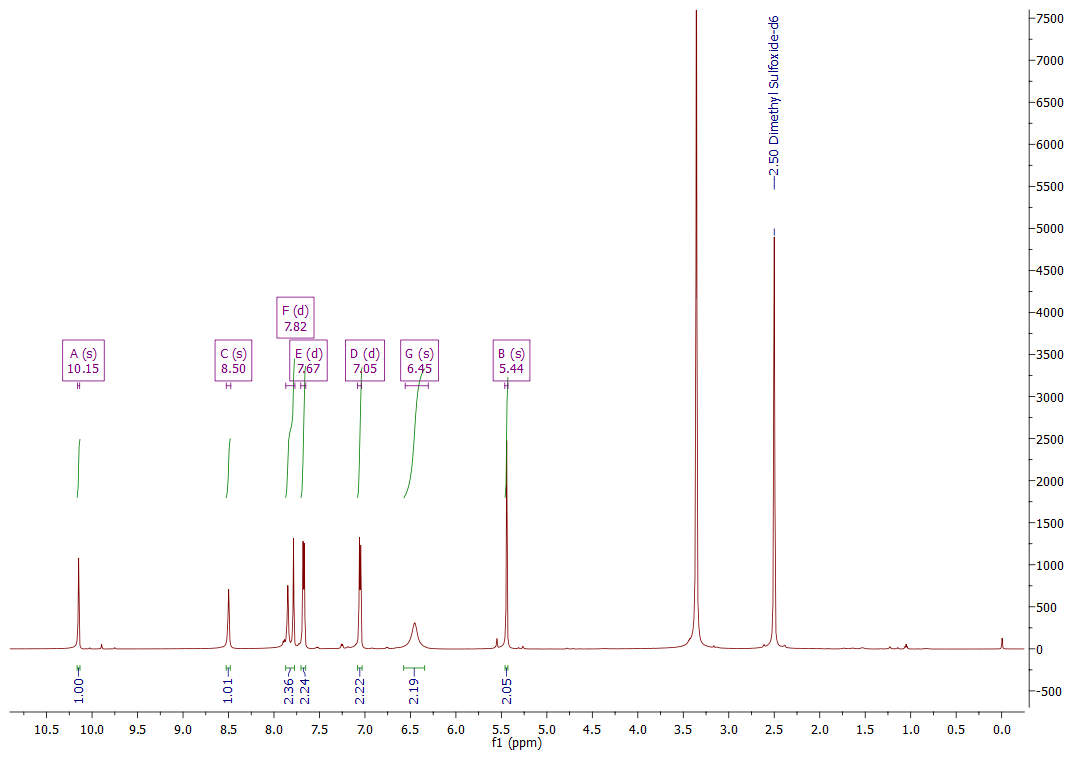
**

**Fig 28.** ^13^C NMR spectrum (151 MHz, DMSO-d6) of compound **4o**

**
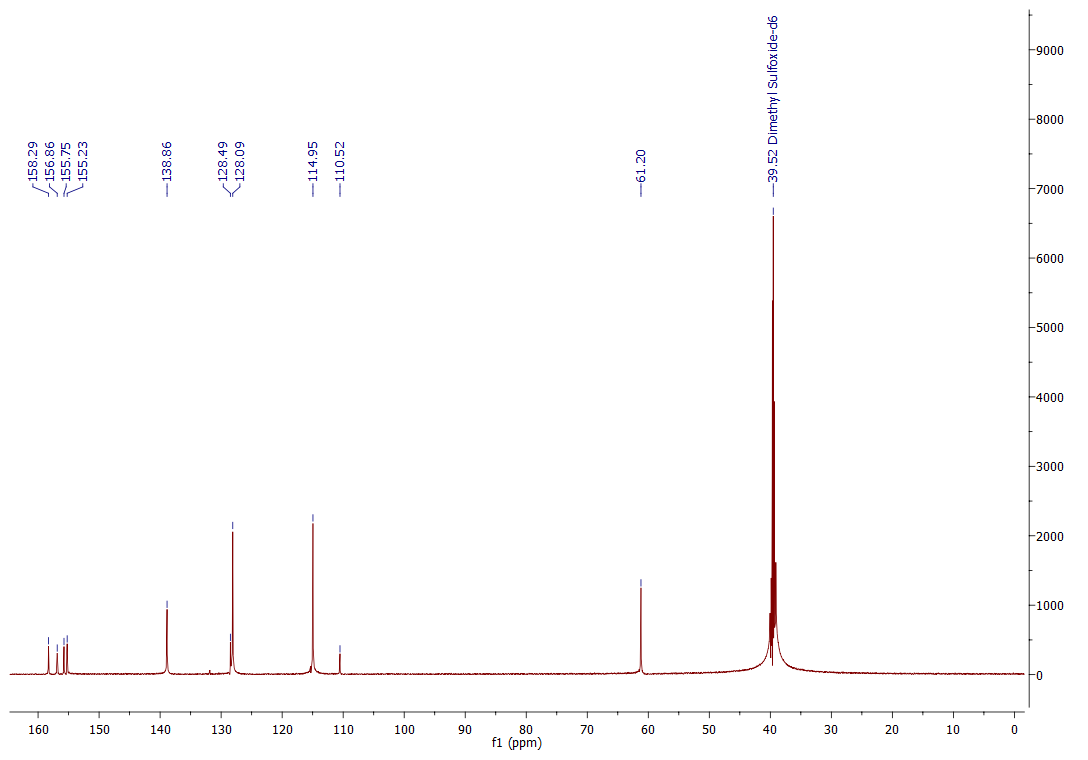
**

**Fig 29.** ^1^H NMR spectrum (600 MHz, DMSO-d6) of compound **14a**

**
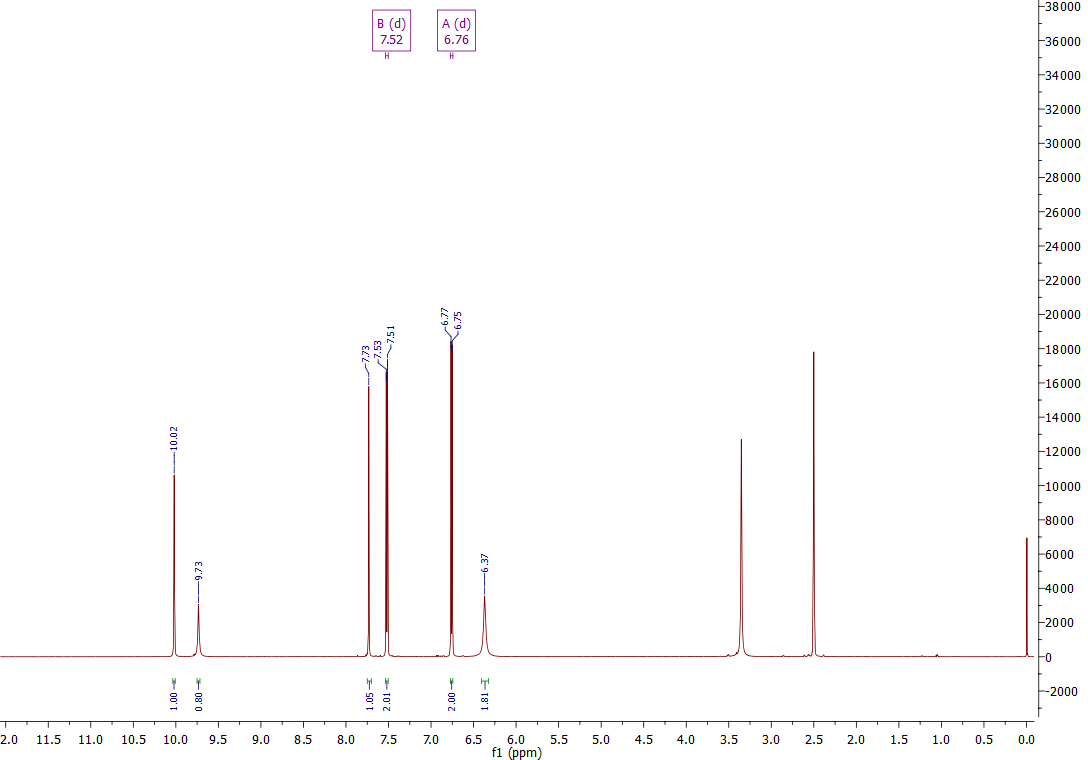
**

**Fig 30.** ^13^C NMR spectrum (151 MHz, DMSO-d6) of compound **14a**

**
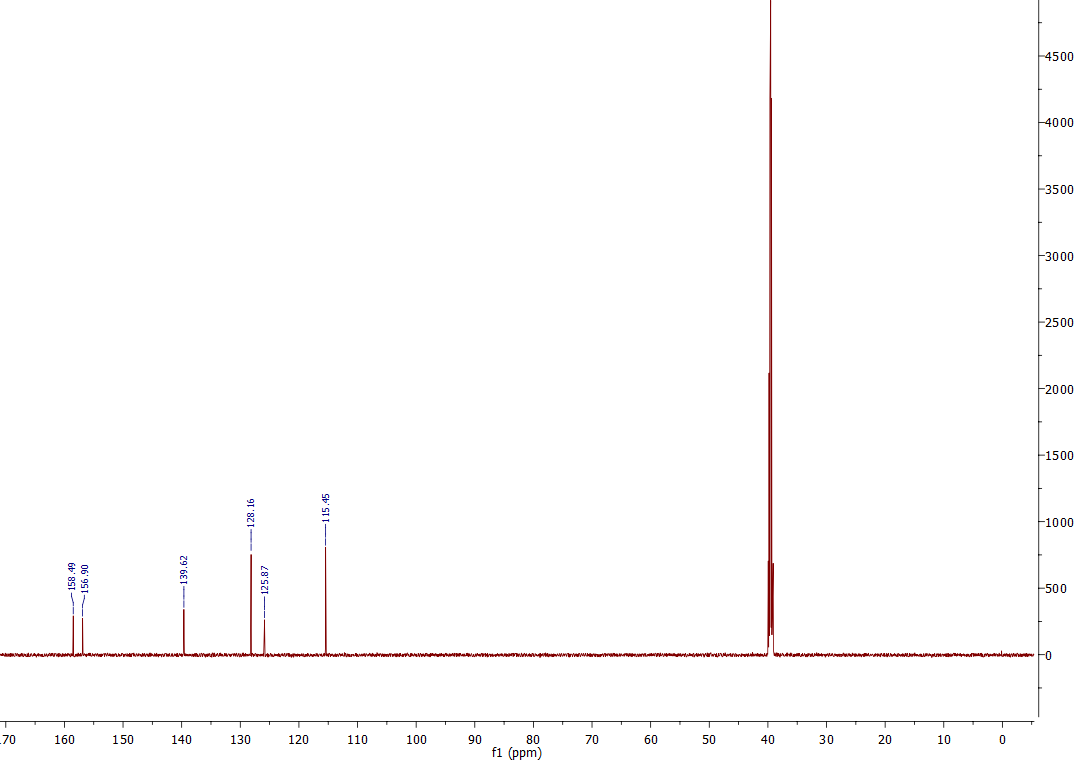
**

**Fig 31.** ^1^H NMR spectrum (600 MHz, DMSO-d6) of compound **14b**

**
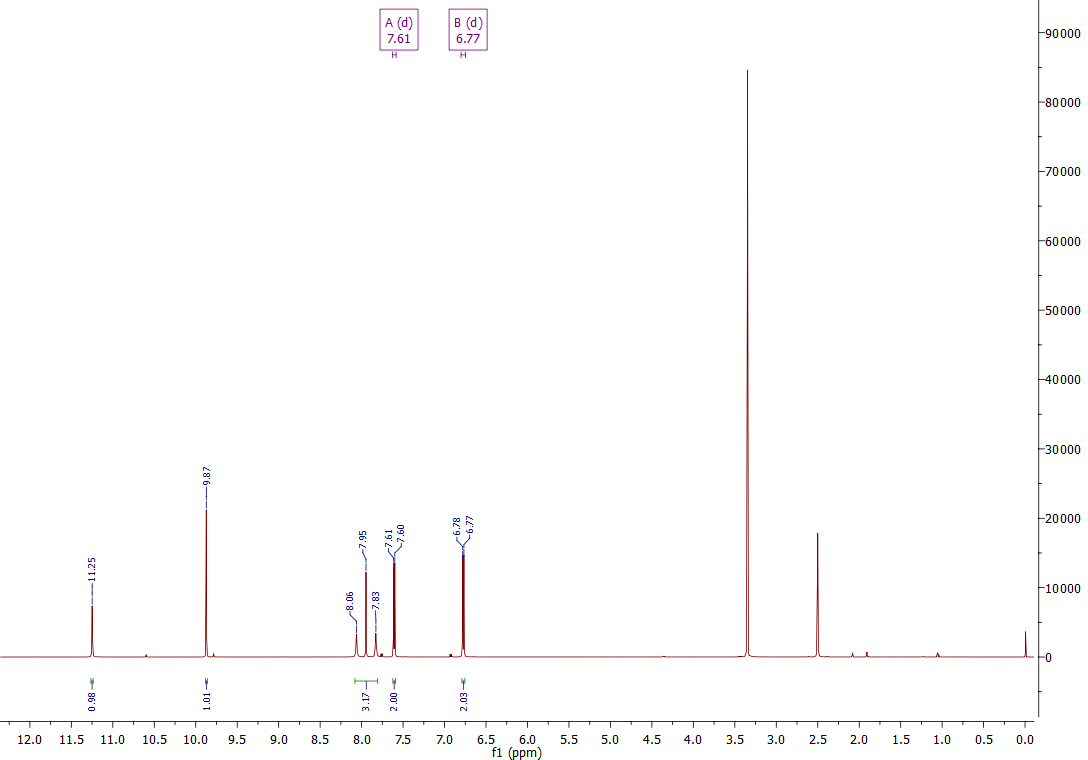
**

**Fig 32.** ^13^C NMR spectrum (151 MHz, DMSO-d6) of compound **14b**

**
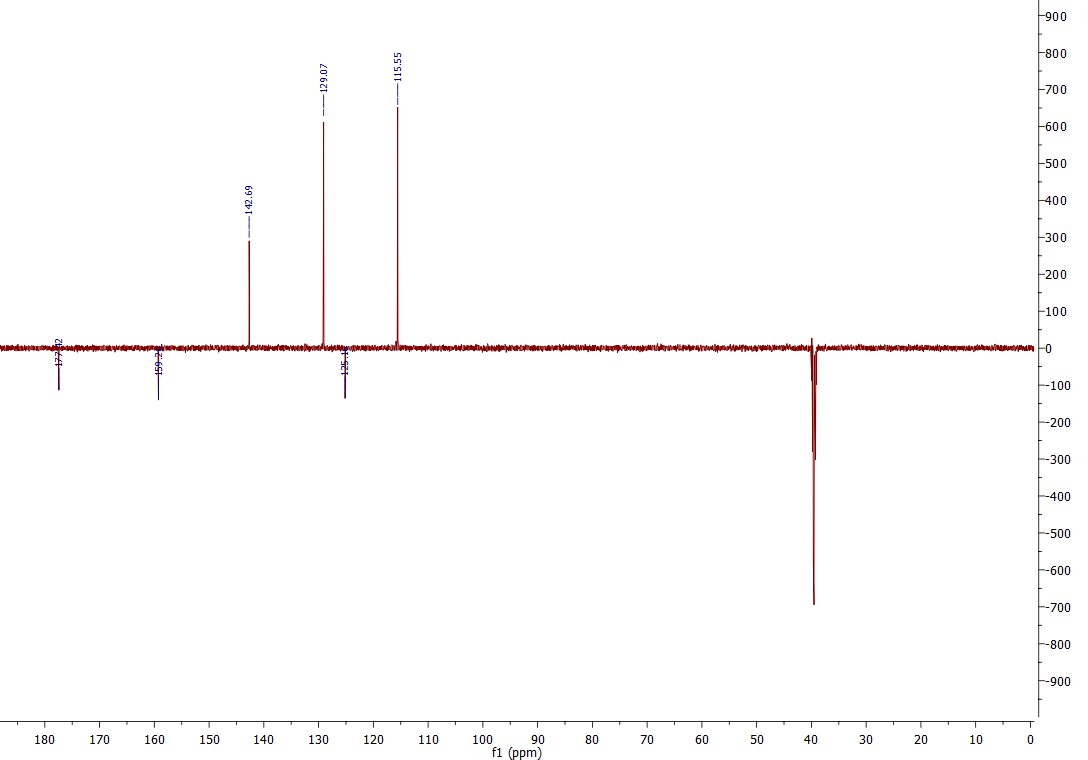
**

**Fig 33.** ^1^H NMR spectrum (600 MHz, DMSO-d6) of compound **14c**

**
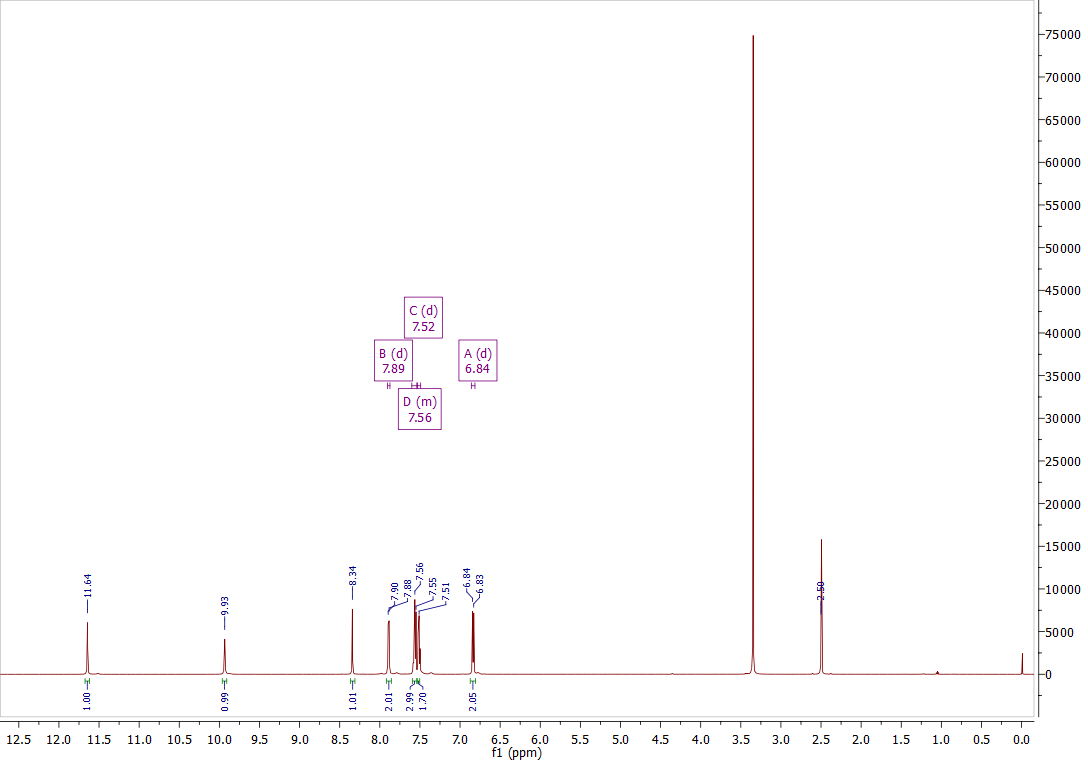
**

**Fig 34.** ^13^C NMR spectrum (151 MHz, DMSO-d6) of compound **14c**

**
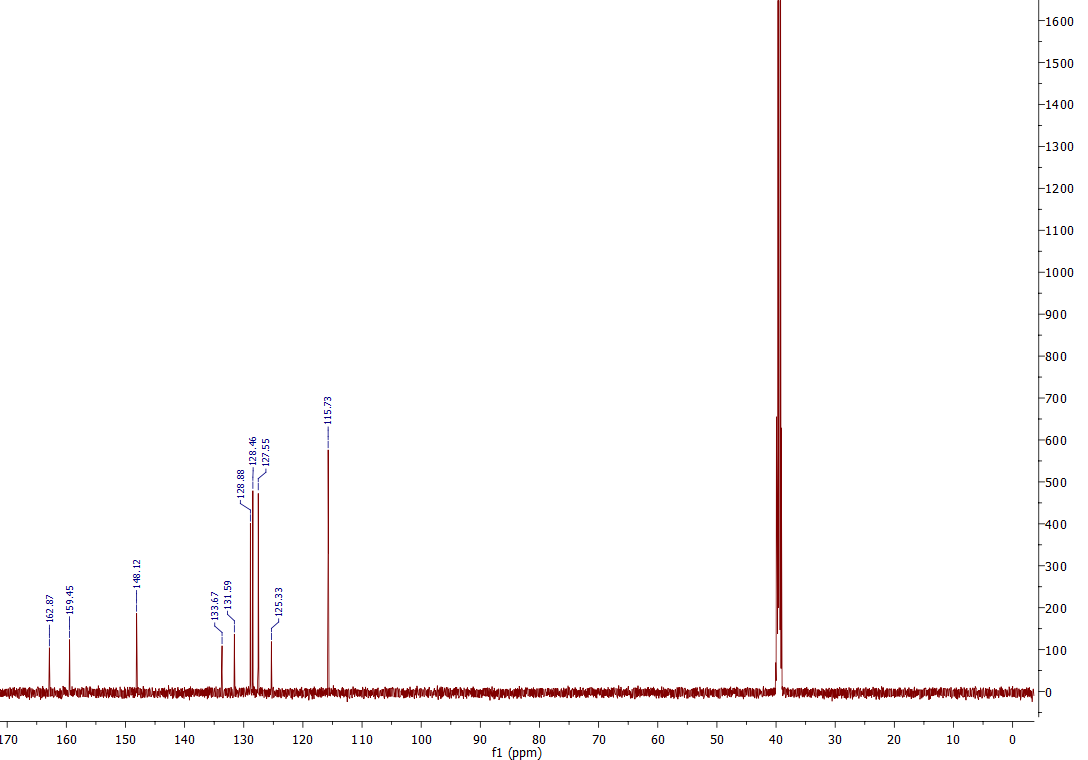
**

**Fig 35.** ^1^H NMR spectrum (600 MHz, DMSO-d6) of compound **14d**

**
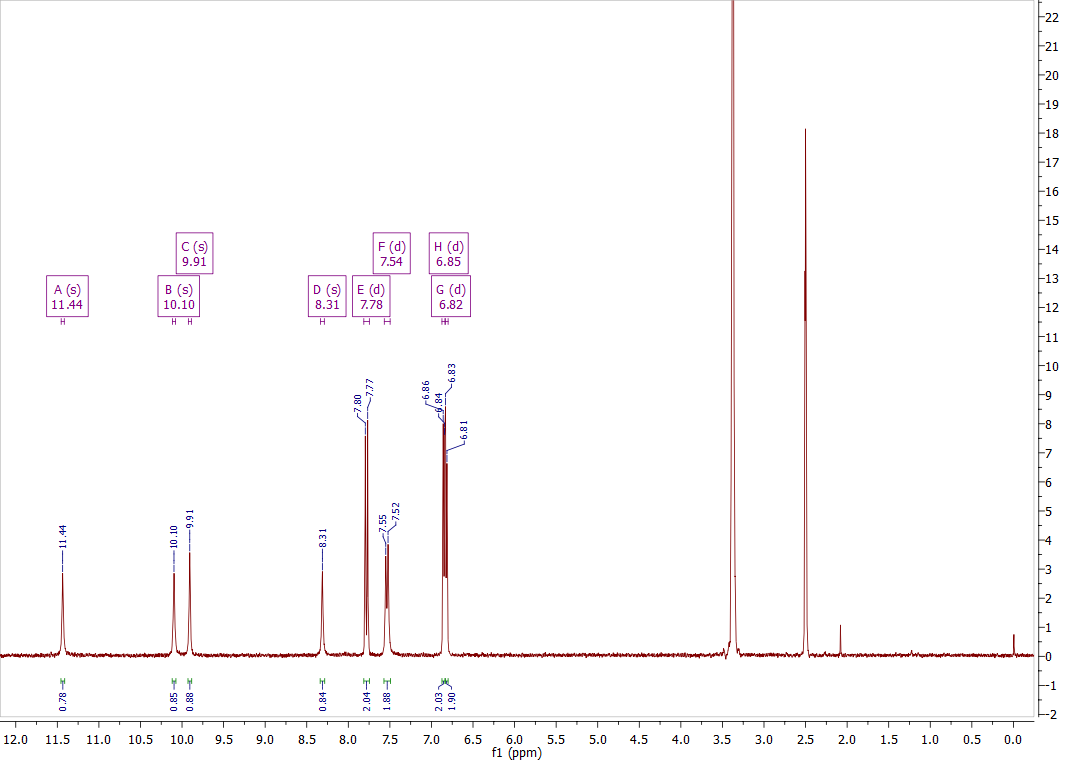
**

**Fig 36.** ^13^C NMR spectrum (151 MHz, DMSO-d6) of compound **14d**

**
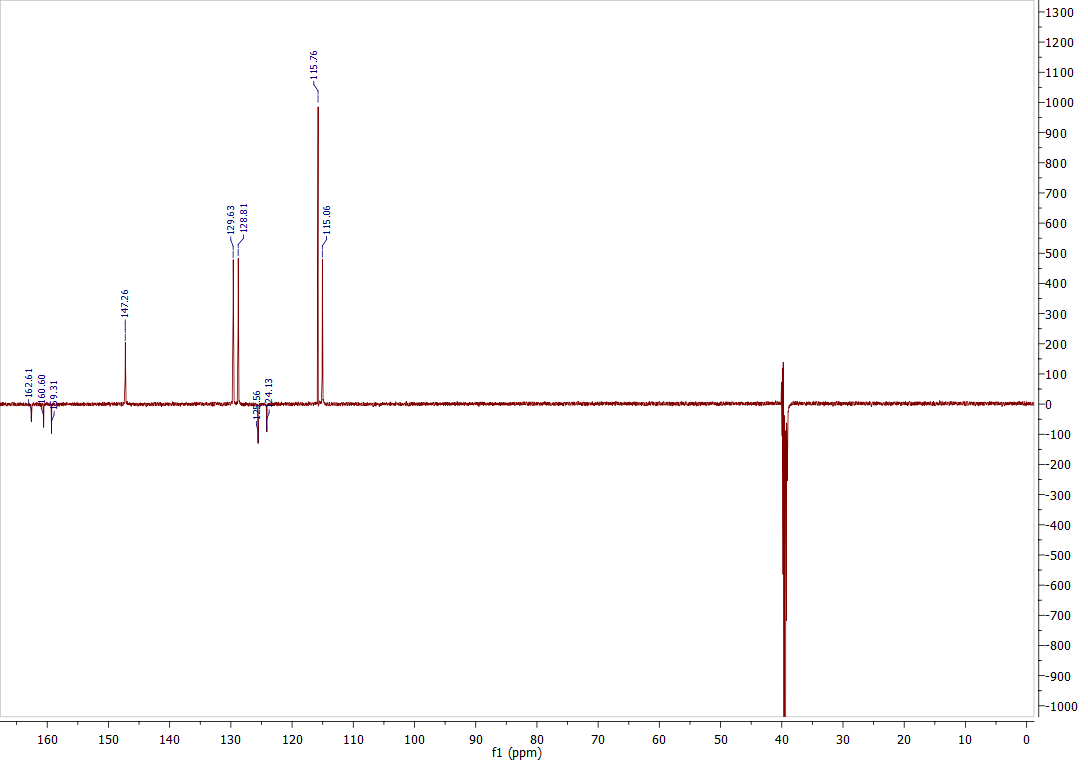
**

**Fig 37.** ^1^H NMR spectrum (600 MHz, DMSO-d6) of compound **14e**

**
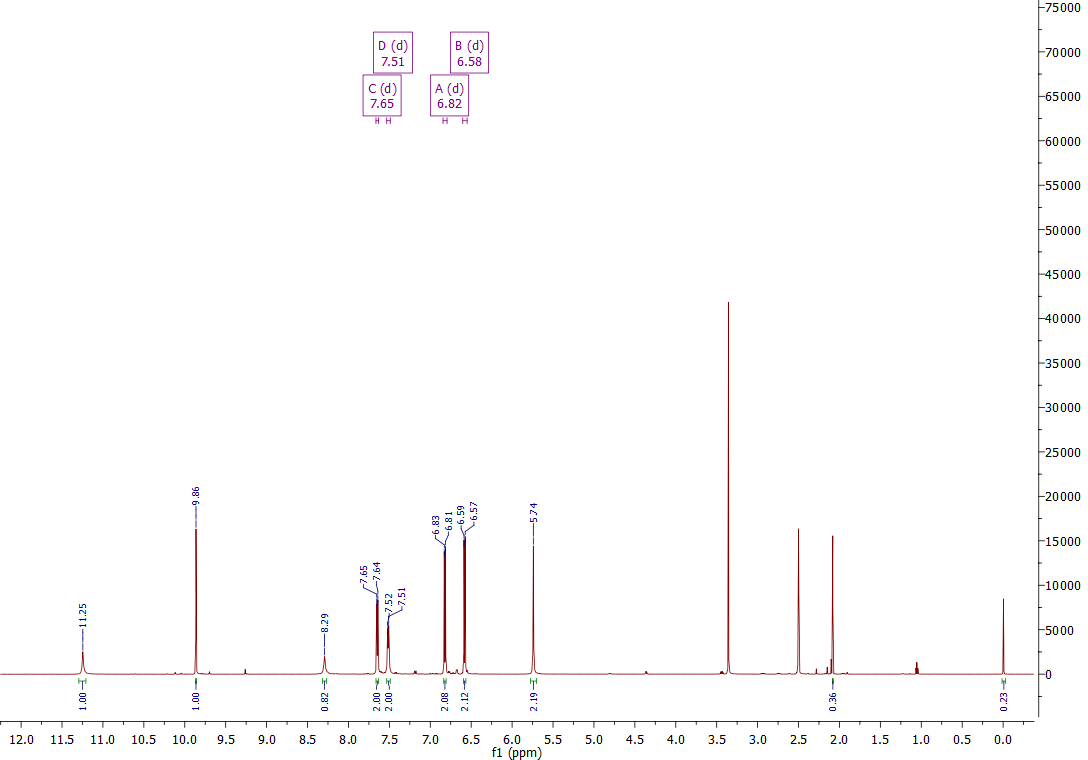
**

**Fig 38.** ^13^C NMR spectrum (151 MHz, DMSO-d6) of compound **14e**

**
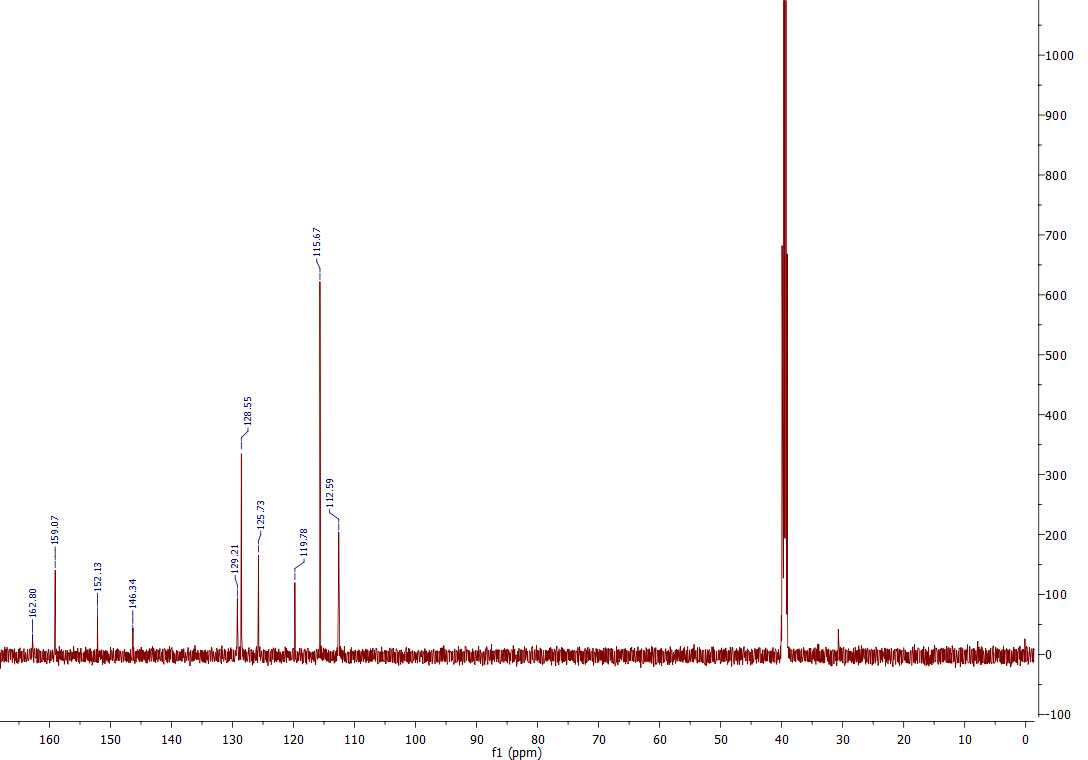
**

**Fig 39.** ^1^H NMR spectrum (600 MHz, DMSO-d6) of compound **14f**

**
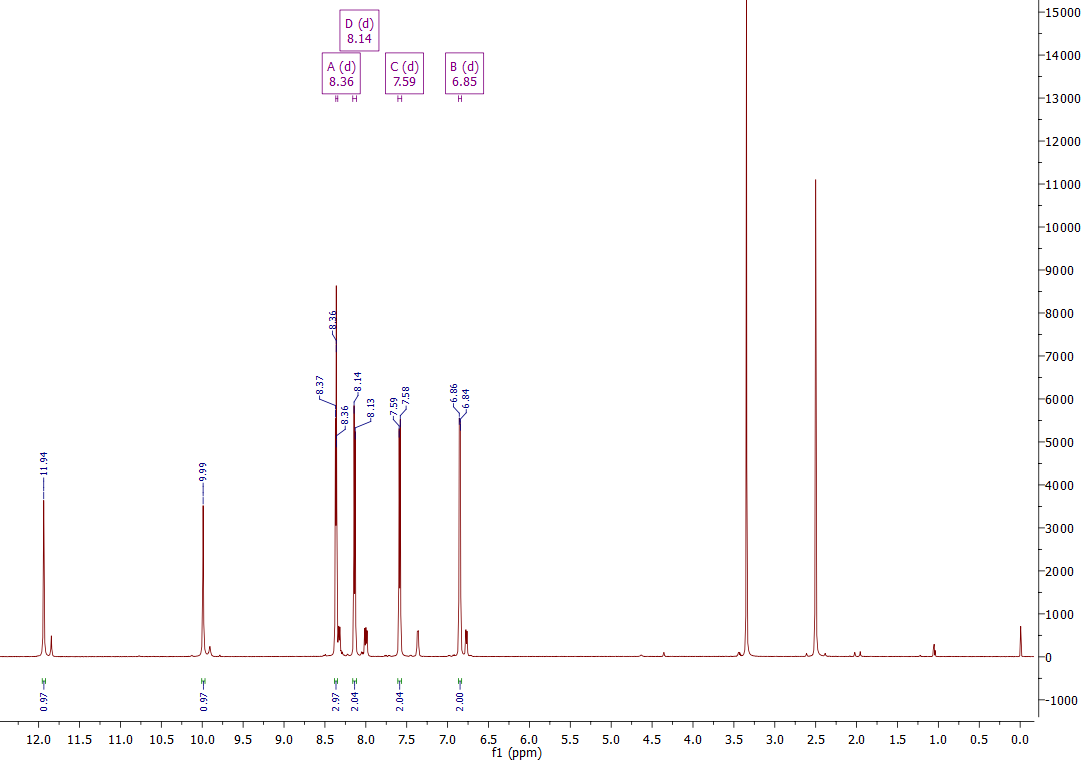
**

**Fig 40.** ^13^C NMR spectrum (151 MHz, DMSO-d6) of compound **14f**

**
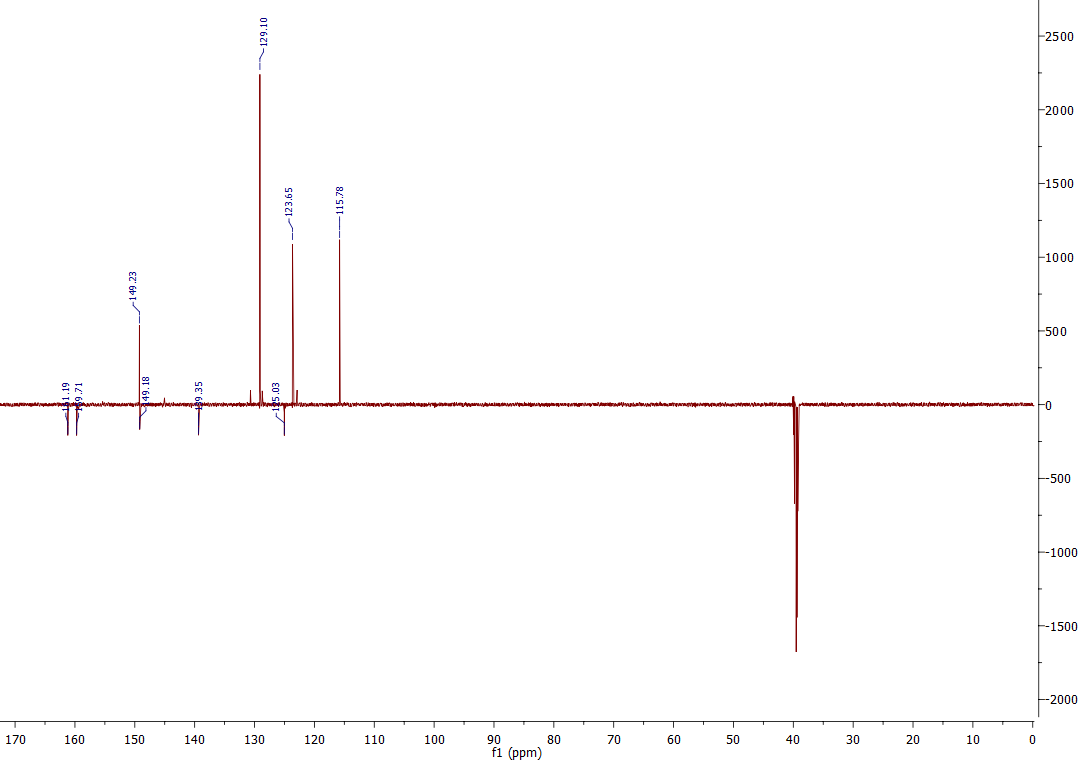
**

**Fig 41.** ^1^H NMR spectrum (600 MHz, DMSO-d6) of compound **14g**

**
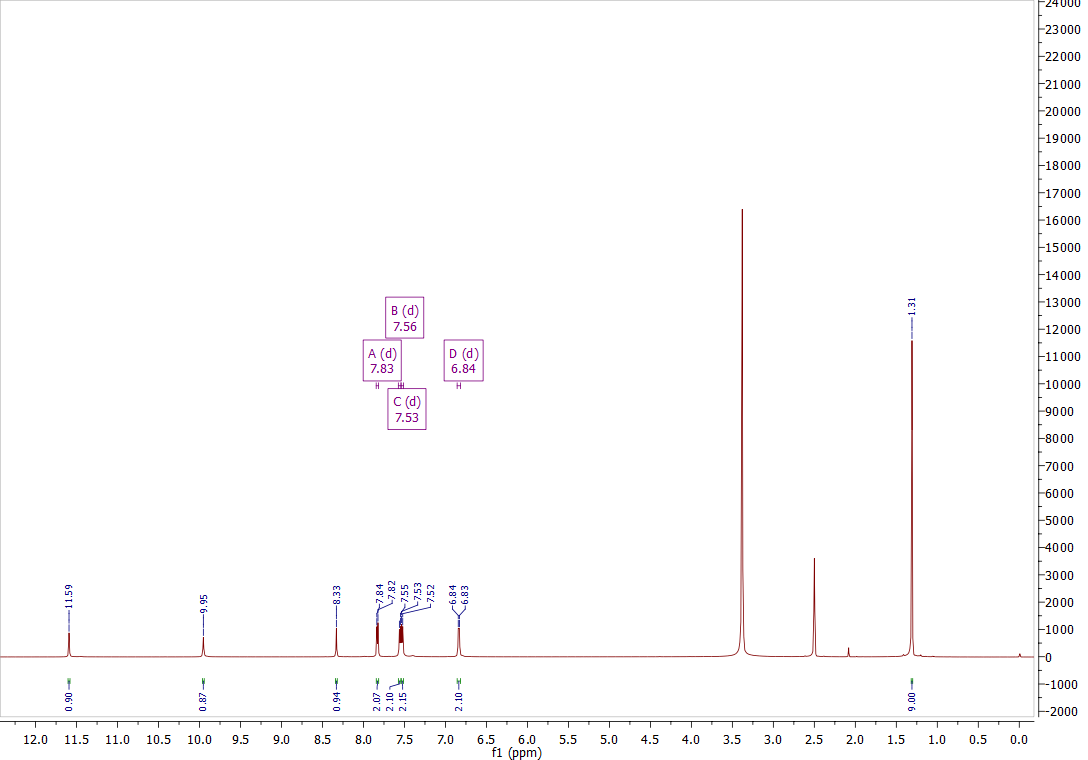
**

**Fig 42.** ^13^C NMR spectrum (151 MHz, DMSO-d6) of compound **14g
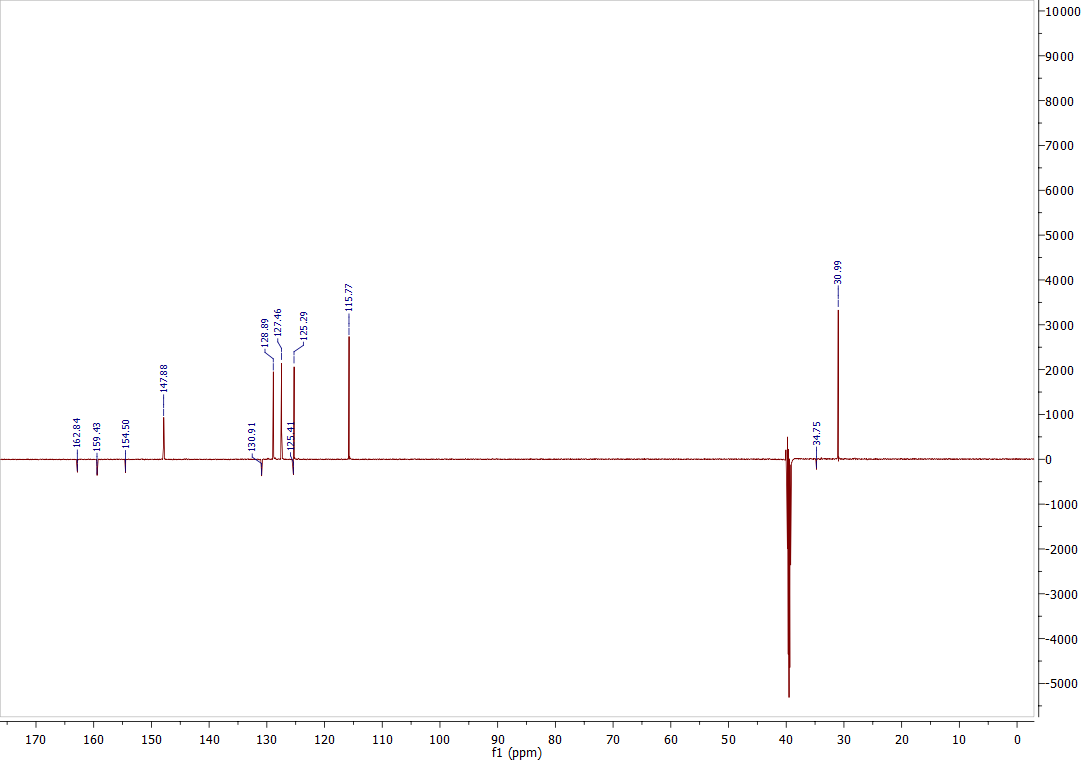
**
