# Supplementary material for: Design, synthesis and biological evaluation of N-oxide derivatives with potent in vivo antileishmanial activity
Source: PLoS One. 2021 Nov 1;16(11):e0259008. doi: 10.1371/journal.pone.0259008 (PMC8559926; doi:10.1371/journal.pone.0259008)
Supplement: S1 Table — (DOCX) [file pone.0259008.s002.docx]

| **Physicochemical properities** | **Calculated values** | | | | | | | | | |  |
| --- | --- | --- | --- | --- | --- | --- | --- | --- | --- | --- | --- |
|  | **4a** | **4b** | **4c** | **4d** | **4e** | **4f** | **4g** | **4h** | **4l** | **4m** | **4r** |
| Num. heavy atoms | 30 | 31 | 31 | 33 | 34 | 25 | 25 | 28 | 31 | 32 | 25 |
| Num. arom. heavy atoms | 23 | 23 | 23 | 23 | 23 | 17 | 17 | 17 | 17 | 17 | 15 |
| Fraction Csp^3^ | 0 | 0 | 0 | 0 | 0.15 | 0 | 0 | 0.06 | 0.06 | 0.23 | 0.22 |
| Num. rotatable bonds | 7 | 7 | 7 | 8 | 8 | 6 | 6 | 8 | 9 | 9 | 5 |
| Num. H bond receptors | 6 | 7 | 6 | 8 | 6 | 6 | 5 | 7 | 9 | 7 | 5 |
| Num. H bond donors | 1 | 2 | 2 | 1 | 1 | 2 | 2 | 2 | 2 | 2 | 1 |
| *Lipophilicity* | | | | | | | | | | | |
| Log P_o/w_ (iLOGP) | 2.87 | 2.54 | 2.4 | 2.21 | 3.91 | 1.33 | 1.77 | 0.81 | 0.01 | 2.03 | 2.29 |
| *Solubility* | | | | | | | | | | | |
| Water solubility (mg/mL)(ESOL) | 1.11 e-3 | 1.59 e-3 | 2.59 e-3 | 2.95 e-3 | 4.74e-5 | 3.41 e-1 | 1.98 e-2 | 5.60 e-2 | 1.48 e-1 | 3.93 e-3 | 4.41 e-3 |
| class | Moderately soluble | Moderately soluble | Moderately soluble | Moderately soluble | Poorly soluble | Soluble | Moderately soluble | Soluble | Soluble | Moderately soluble | Moderately soluble |
| *Pharmacokinetics* | | | | | | | | | | | |
| GI absorption | High | High | Low | Low | Low | High | Low | Low | Low | Low | High |
| BBB permeant | No | No | No | No | No | No | No | No | No | No | No |
| P-gp substrate | No | No | No | No | Yes | No | No | No | Yes | No | No |
| CYP1A2 inhibitor | Yes | Yes | No | Yes | No | Yes | Yes | No | No | No | No |
| CYP2C19 inhibitor | Yes | No | Yes | Yes | Yes | No | Yes | Yes | Yes | Yes | No |
| CYP2C9 inhibitor | No | No | No | No | Yes | No | No | No | No | No | No |
| CYP2D6 inhibitor | No | No | No | No | No | No | No | No | No | No | No |
| CYP3A4 inhibitor | No | No | No | No | No | No | No | No | No | No | No |
| Log *kp* (skin permeation) | -5.21 cm/s | -5.56 cm/s | -5.79 cm/s | -6.10 cm/s | -4.37 cm/s | -6.42 cm/s | -6.10 cm/s | -6.10 cm/s | -7.59 cm/s | -5.86 cm/s | -5.14 cm/s |
| *Druglikeness* | | | | | | | | | | | |
| Lipinski | Yes; | Yes | Yes | Yes; 1 violation | Yes, 1 violation | Yes | Yes | Yes; | Yes; 1 violation | Yes; 0 violation | Yes; 0 violation |
| Ghose | Yes | Yes | Yes | Yes | No | Yes | Yes | Yes | Yes | Yes | Yes |
| Veber | Yes | Yes | Yes | No; 1 violation | Yes | Yes | No; 1 violation | No; 1 violation | No; 1 violation | No; 1 violation | Yes |
| Egan | Yes | Yes | Yes | No; 1 violation | Yes | Yes | No; 1 violation | No; 1 violation | No; 1 violation | No; 1 violation | Yes |
| Muegge | Yes | Yes | Yes | No; 1 violation | No | Yes | Yes | Yes | No; 1 violation | Yes | Yes |
| Bioavailability Score | 0.56 | 0.56 | 0.56 | 0.11 | 0.56 | 0.56 | 0.56 | 0.56 | 0.11 | 0.56 | 0.56 |
| *Medicinal chemistry* | | | | | | | | | | | |
| PAINS alert | 0 | 0 | 0 | 0 | 0 | 0 | 0 | 0 | 0 | 0 | 0 |
| Synthetic accessibility | 3.83 | 3.87 | 3.83 | 3.87 | 4.22 | 3.65 | 3.66 | 3.55 | 3.64 | 3.95 | 3.42 |

**Pharmacokinetics:** High and Low indicates gastrointestinal absorption; Yes and No indicates BBB (Blood Brain Barrier) permeation or P-gp (glycoprotein P). **Druglikeness:** Yes and No indicates that the molecule is according to all Lipinski, Ghose, Veber, Egan or Muegge descritors. **Bioavailability Score**: Predict the probability of a compound to have at least 10% oral bioavailability in rat or measurable Caco-2 permeability, calculating the probalitities of 11%, 17%, 56% or 85%.
