# Supplementary figures and images for: Design, synthesis and biological evaluation of N-oxide derivatives with potent in vivo antileishmanial activity
Source: PLoS One. 2021 Nov 1;16(11):e0259008. doi: 10.1371/journal.pone.0259008 (PMC8559926; doi:10.1371/journal.pone.0259008)

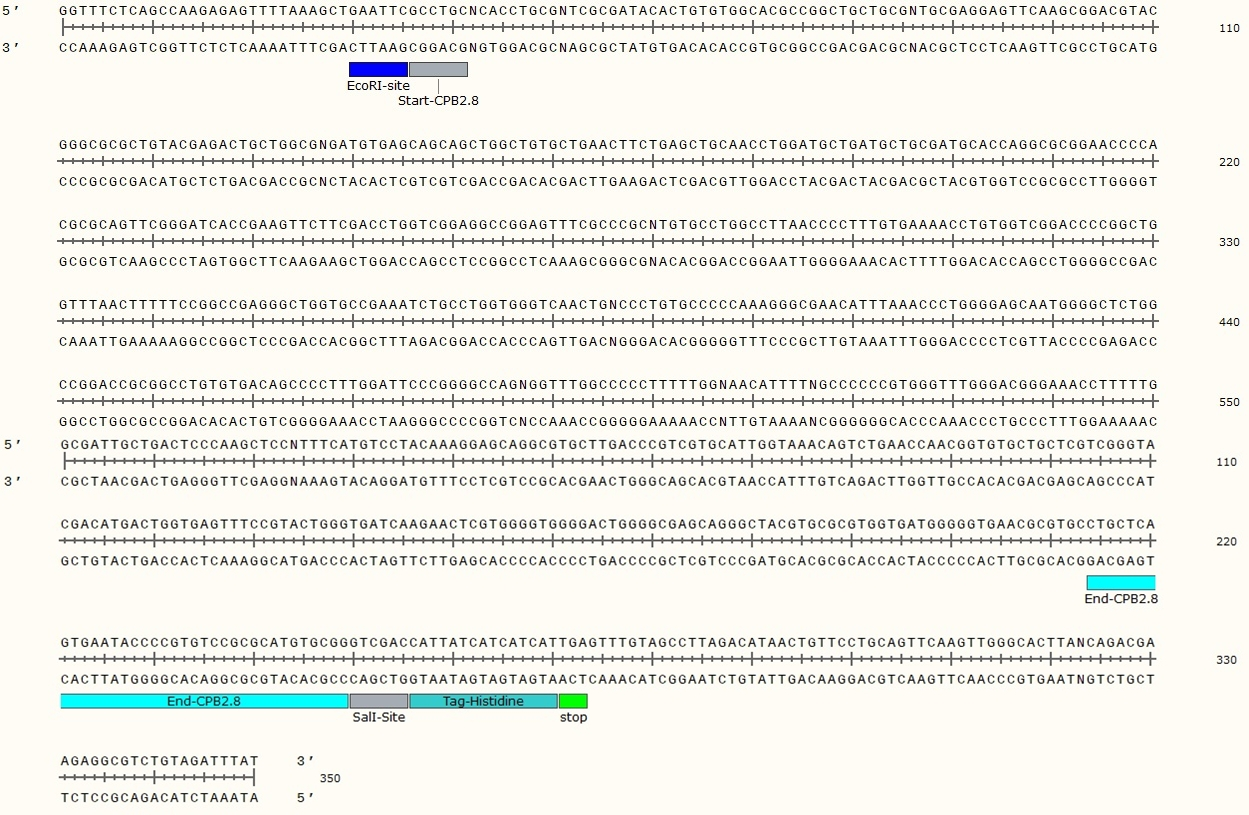

Supplement: S1 Fig — The clone was sequenced using α factor (TACTATTGCCAGCATTGCTGC) and reverse 3’AOX1(GCAAATGGCATTCTGACATCC), and the elements inserted on plasmid (EcoRI—blue and Sal I–grey, sites) were identified upstream and downstream of CPB2.8 gene, respectively. (TIF) [file pone.0259008.s003.tif]

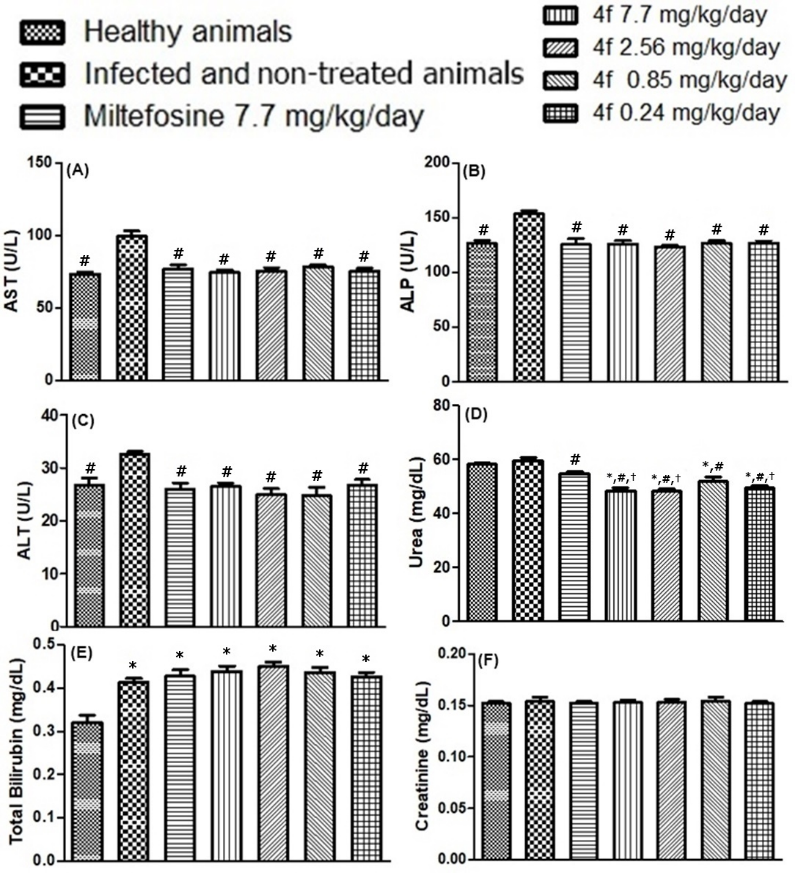

Supplement: S2 Fig — Levels of (A) AST—aspartate aminotransferase; (B) ALP—alkaline phosphatase levels; (C) ALT—alanine aminotransferase; (D) Urea; (E) Total bilirubin levels and (F) Creatinine. The data are expressed as average ± SEM. #: Statistically significant compared to the Infected and Untreated animals (p < 0.05). *: Statistically significant compared to the healthy animals (p < 0.05). †: Statistically significant compared to the animals treated with the reference drug miltefosine (p < 0.05). No statistical significance was observed for creatinine compared to healthy and Untreated animals. (TIF) [file pone.0259008.s004.tif]
