# Supplementary material for: Design, synthesis and biological evaluation of N-oxide derivatives with potent in vivo antileishmanial activity
Source: PLoS One. 2021 Nov 1;16(11):e0259008. doi: 10.1371/journal.pone.0259008 (PMC8559926; doi:10.1371/journal.pone.0259008)
Supplement: S1 Raw images — (PDF) [file pone.0259008.s005.pdf]

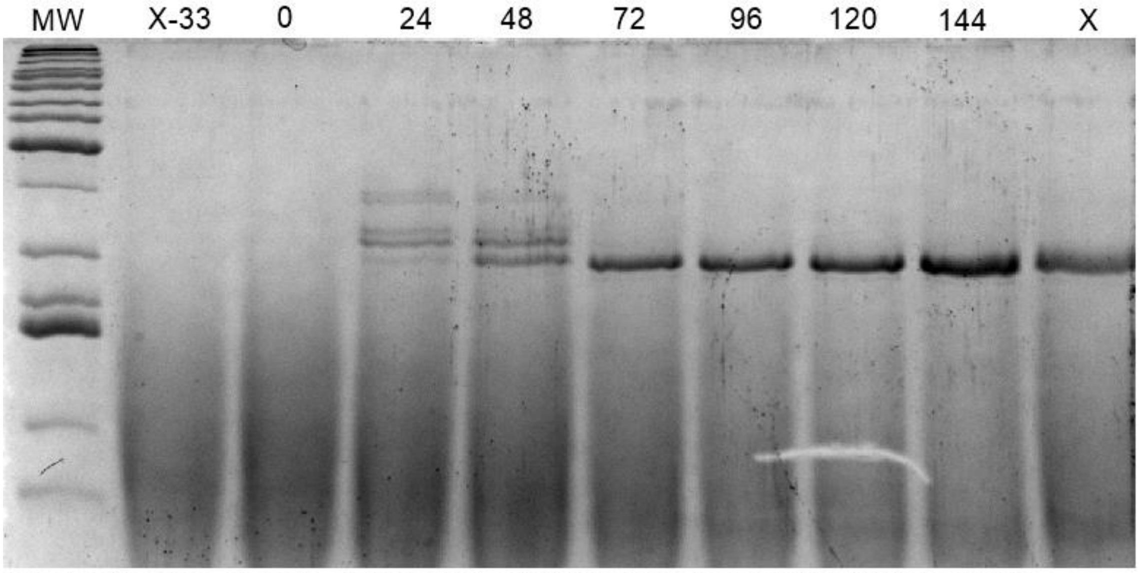

Original file of the Analysis of expression of CPB in P. pastoris. SDS-PAGE showing the supernatants of untransformed Pichia pastoris X-33, and aliquots from 0 to 144 hours of clone pPIC-CPB2.8#25 induced with methanol. MW: molecular weight Bench Mark (Invitrogen) X: unused lane that corresponds to a repetition of 144 h supernatant's aliquot.

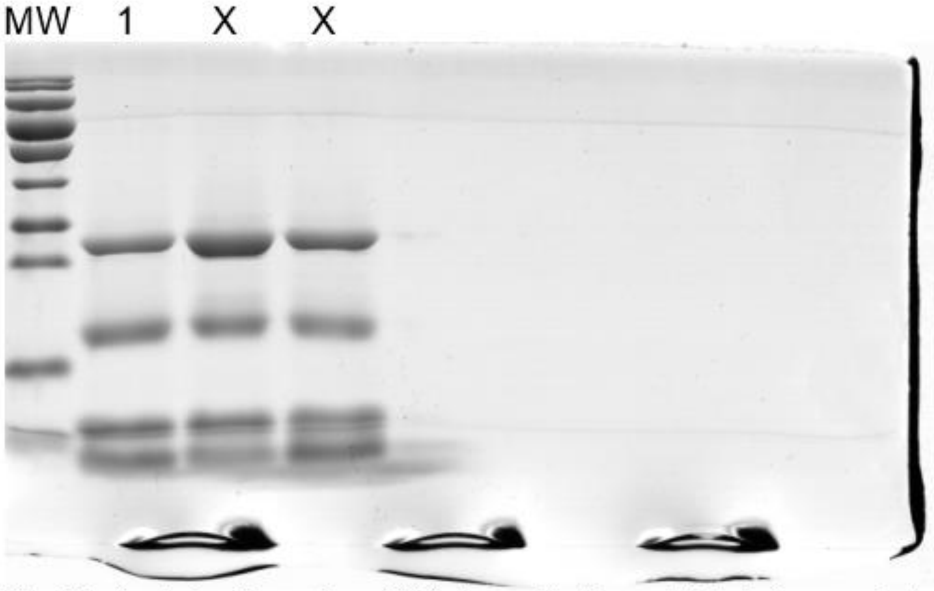

Original file of the Analysis of L. mexicana CPB after purification and dialysis.Coomassie-stained SDS-PAGE 15% showing in MW: molecular weight Page Ruler (Thermo Scientific); 1, the purified and dialyzed rCPB2.8deltaCTE. The recombinant protein (about 26 kDa) and the products of proteolysis. X, unused lanes corresponds to the other samples not used of CPB.
