# Supplementary material for: Design, synthesis and biological evaluation of N-oxide derivatives with potent in vivo antileishmanial activity
Source: PLoS One. 2021 Nov 1;16(11):e0259008. doi: 10.1371/journal.pone.0259008 (PMC8559926; doi:10.1371/journal.pone.0259008)
Supplement: Attachment — Submitted filename: Response to Reviewers (1).docx [file pone.0259008.s006.docx]

**We´d like to thank the reviewers for use your precious time, giving us new feedback that certainly helped us to improve the quality manuscript. We´d like also to wish you all to be safe during this difficult time of social distance cause by SARS-CoV-2. Whenever is possible, stay at home.**

**Comments from the editor:**

1. Please ensure that your manuscript meets PLOS ONE's style requirements, including those for file naming. The PLOS ONE style templates can be found at

<https://journals.plos.org/plosone/s/file?id=wjVg/PLOSOne_formatting_sample_main_body.pdf> and

<https://journals.plos.org/plosone/s/file?id=ba62/PLOSOne_formatting_sample_title_authors_affiliations.pdf>

**Author’s response:** Thank you for the recommendation. The manuscript was revised according PLOS ONE guideline for authors.

In reference to the itens 2, 3 and 4

2. We note that the grant information you provided in the ‘Funding Information’ and ‘Financial Disclosure’ sections do not match.

When you resubmit, please ensure that you provide the correct grant numbers for the awards you received for your study in the ‘Funding Information’ section.

3. Thank you for stating the following financial disclosure:

“NO”

At this time, please address the following queries:

a)        Please clarify the sources of funding (financial or material support) for your study. List the grants or organizations that supported your study, including funding received from your institution.

b)        State what role the funders took in the study. If the funders had no role in your study, please state: “The funders had no role in study design, data collection and analysis, decision to publish, or preparation of the manuscript.”

c)        If any authors received a salary from any of your funders, please state which authors and which funders.

d)        If you did not receive any funding for this study, please state: “The authors received no specific funding for this work.”

Please include your amended statements within your cover letter; we will change the online submission form on your behalf.

4. Thank you for stating the following in the Acknowledgments Section of your manuscript:

“This study was supported by Fundação de Amparo à Pesquisa do Estado de São Paulo (FAPESP Process: 2016/09502-7, 2016/06931-4, 2018/17739-2, 2018/11079-0 and 2017/03552-5). Programa de Apoio ao Desenvolvimento Científico da Faculdade de Ciências Farmacêuticas da UNESP – PADC. This study was financed in part by the Coordenação de Aperfeiçoamento de Pessoal de Nível Superior - Brasil (CAPES) and Conselho Nacional de Desenvolvimento Científico e Tecnológico (CNPq). L.C.C. is CNPq PhD student fellow (143334/2017-4). I.M.P is CNPq postdoctoral fellow (153581/2018-2). J.L.S, F.H.S. and M.A.S.G are recipient of a Research Productivity Scholarship from the National Council for Research and Development (CNPq Ref. Process: 304731/2017-0, 311746/2017-9 and 305174/2020-7, respectively). The authors are grateful to Profº André Tempone and Felipe Torres for Leishmania infantum (MHOM/MA67IMA263) donation. The funders had no role in study design, data collection and analysis, decision to publish, or preparation of the manuscript.”

We note that you have provided additional information within the Acknowledgements Section that is not currently declared in your Funding Statement. Please note that funding information should not appear in the Acknowledgments section or other areas of your manuscript. We will only publish funding information present in the Funding Statement section of the online submission form.

Please remove any funding-related text from the manuscript and let us know how you would like to update your Funding Statement. Currently, your Funding Statement reads as follows:

“MASG (2017/03552-5 and 2016/06931-4) FAPESP - The São Paulo Research Poundation and (305174/2020-7) CNPq - National Council for Scientific and Technological Development

JLS (2018/11079-0) FAPESP - The São Paulo Research Poundation and (304731/2017-0) CNPq - National Council for Scientific and Technological Development

FHS (311746/2017-9) CNPq - National Council for Scientific and Technological Development

LCC (143334/2017-4) CNPq - National Council for Scientific and Technological Development

IMP (153581/2018-2) CNPq - National Council for Scientific and Technological Development

GFSF (2016/095502-7) (2018/17739-2) - FAPESP - The São Paulo Research Foundation

CNPq: https://www.gov.br/cnpq/pt-br

FAPESP: https://fapesp.br/en/about

Did the sponsors or funders play any role in the study design, data collection and analysis, decision to publish, or preparation of the manuscript?

NO.”

Please include your amended statements within your cover letter; we will change the online submission form on your behalf.

**Author's response to critics/suggestion 2, 3 and 4:**

Thank you for all the critiques/suggestions. We have reviewed all financial information as well as removed it from the Acknowledgement section of the manuscript, according to the "submission guidelines''.

We'd like to kindly ask you to change the online submission form on our behalf using the below reviewed funding information:

"We are grateful to The São Paulo Research Foundation, FAPESP, <https://fapesp.br>/en, (2017/03552-5, 2016/06931-4 and 2018/11079-0) and National Council for Scientific and Technological Development, CNPq, https://www.gov.br/cnpq/pt-br, (305174/2020-7, 304731-2017-0 and 311746/2017-9) for financial support. The funders had no role in study design, data collection and analysis, decision to publish, or preparation of the manuscript."

5. PLOS ONE now requires that authors provide the original uncropped and unadjusted images underlying all blot or gel results reported in a submission’s figures or Supporting Information files. This policy and the journal’s other requirements for blot/gel reporting and figure preparation are described in detail at https://journals.plos.org/plosone/s/figures#loc-blot-and-gel-reporting-requirements and https://journals.plos.org/plosone/s/figures#loc-preparing-figures-from-image-files. When you submit your revised manuscript, please ensure that your figures adhere fully to these guidelines and provide the original underlying images for all blot or gel data reported in your submission. See the following link for instructions on providing the original image data: https://journals.plos.org/plosone/s/figures#loc-original-images-for-blots-and-gels.

In your cover letter, please note whether your blot/gel image data are in Supporting Information or posted at a public data repository, provide the repository URL if relevant, and provide specific details as to which raw blot/gel images, if any, are not available. Email us at plosone@plos.org if you have any questions.

**Author’s response:** Thank you for the recommendation. The gels images used in the manuscript (Fig 2A and B) are also shown uncropped and unadjusted in the "supplementary material" named (S1_raw_images).

6. Please review your reference list to ensure that it is complete and correct. If you have cited papers that have been retracted, please include the rationale for doing so in the manuscript text, or remove these references and replace them with relevant current references. Any changes to the reference list should be mentioned in the rebuttal letter that accompanies your revised manuscript. If you need to cite a retracted article, indicate the article’s retracted status in the References list and also include a citation and full reference for the retraction notice.

**Author’s response:** Thank you for the recommendation. The references were carefully revised according to the PLos One guidelines. In fact, two missing references were now added (lines 809-815):

30.	Isabel TF, Costa GNM, Pacheco IB, Barbosa LG, Santos-Junior CD, Fonseca FPP, et al. Expression and partial biochemical characterization of a recombinant serine protease from Bothrops pauloensis snake venom. Toxicon. 2016. doi:10.1016/j.toxicon.2016.03.002

31.	Boettner M, Prinz B, Holz C, Stahl U, Lang C. High-throughput screening for expression of heterologous proteins in the yeast Pichia pastoris. J Biotechnol. 2002. doi:10.1016/S0168-1656(02)00157-8

Additionally, two other references were also revised (lines 774-776 and 832-836 respectively):

20.	de Almeida L. Leishmanioses e derivados de furoxano e benzofuroxano : atividade biológica in vitro e in vivo e potenciais mecanismos de ação. Universidade Estadual Paulista “Júlio de Mesquita Filho” [Doctoral Thesis]. Araraquara, 2017.

37.	Velasquez AMA. Do screening ao mecanismo de ação, uma contribuição para a descoberta de ciclopaladados bioativos: a atividade leishmanicida de CP2 e seu efeito inibitório frente à DNA topoisomerase 1B de Leishmania. Universidade Estadual Paulista “Júlio de Mesquita Filho” [Doctoral Thesis]. Araraquara; 2017.

Additional Editor Comments (if provided):

The manuscript reports synthesis of 24 compounds which are tested on Leishmania-infected BALB/c mice for their anti-leishmanial activities. The identified one compound show significant anti-leishmanial activity in both spleen and liver.

However, the manuscript requires several moderations as follows:
1. Please merge Figures 2, 3 and 4 into one - Figure 2
2. Please merge Figures 5 and 6 into one - Figure 3
3. Please merge Figure 7 and 8 into one - Figure 4
4. Please move Figure 9 into supplementary figure.

This will help shorten the manuscript. Please check the composition of the manuscript.

**Author’s response:** Thank you for the suggestion. We do agree with the editor's suggestion and the figures were merged as follow:

1. Figures 2, 3 and 4 were combined into Figure 2;

2. Figures 5 and 6 were combined into Figure 3;
2. Figures 7 and 8 were combined into Figure 4

Another suggestion related to Figure 9 was also well accepted by all author's and moved to the Supporting information (S2 Fig).

The manuscript was revised after the implemented modifications.

**Reviewer’s comments:**

1. Page 24, Line 566, the figure legend needs to be written more elaborately.

**Author’s response:** Thank you for the suggestion regarding the legend of Figure 4. It is important to inform you that this figure was now part of Figure 2 (resulting from the merge of figures 2, 3 and 4), following the academic editor's suggestion. Please, see below the rewritten legend (lines 519-529):

**Fig 2. Analysis of CPB expression in *P. pastoris*.** (A) SDS-PAGE showing the supernatants of non- transformed *Pichia pastoris* X-33, and aliquots from 0 to 144 hours of the transformed clone pPIC-CPB2.8#25 induced with methanol. The arrow indicates the expected size for active (processed) CPB. The arrowhead indicates the unprocessed protein after 24 and 48 h induction. MW: molecular weight Bench Mark (*Invitrogen*). (**B)** Analysis of *L. mexicana* CPB after purification and dialysis. Coomassie-stained SDS-PAGE 15% showing in MW: molecular weight Page Ruler (Thermo Scientific); 1, the purified and dialyzed recombinant CPB. The arrow indicates the recombinant protein (about 26 kDa) and the arrowheads, the products of proteolysis. (**C)** Active site titration of the recombinant CPB using the irreversible protease inhibitor E-64. Residual activity % (Relative Fluorescence Units) was measured by the ratio between the enzyme activity in absence or presence of increasing concentrations of E-64.

2. Figure Number 5 is unreadable.

**Author’s response:** Thank you for the observation. We do hope that the figure is now clear after the changes made in order to improve its quality. We'd also like to inform you that this figure was merged into Figure 3 (Figure 5 + Figure 6), according to the academic editor's suggestion.

3. Page no 28, line number 601, figure number 6B and 6C is cited. No figure 6B and 6C is in the paper.
4. Page no 28, line no 607, figure 7 is cited, the description does not match with figure7.

**Author’s response:** Thank you for the observation. Indeed, we committed a mistake regarding Figures 6B and 6C. The figure is now correctly cited in the text. We 'd like to call your attention that these figures were merged to another one (Figures 5 + Figure 6 = Figure 3), according to academic editor's suggestion and its citation (now Figure 3) could be seen in (lines 557 -573). In reference to Figure 7, the citation is now corrected.

“The contribution of the *N*-oxide group of the furoxans and benzofuroxans (**4a-x**) was orientated on the S1’ pocket, performing an interaction between a cation-π and the TRP186 residue (Fig 3B and C). Additionally, the imine carbon (C=N) of the *N*-acylhydrazone moiety (**4a-x**) has been positioned near the CYS26 at an average distance of 3.56 Å and the N-H of their scaffolds interacts with ASN163 residue through an H-bond (Fig 3B and C).

Superposition of **14a**, **4a,** and **4e** revealed an occupancy of the S2 pocket by the phenyl ring of the phenylfuroxan derivatives (**4a-g**) (Fig 3). Besides, a hydrophobic interaction between the furoxan **4e** and the S2 pocket**,** not observed for the hydrazone **14a**, might explain the two-fold higher inhibitory enzyme activity exhibited by the furoxan. The improvement on the occupancy by phenylfuroxan derivatives (**4a-g**) in the CPB can also explain the better inhibitory effect of this series compared with its respective hydrazones (**14a-g**) (Fig 3).

**Fig 3.** **Ligand-CPB complex predicted by molecular docking.** Possible poses and interactions performed by compounds **14a (A)**, **4a** **(B)** and **4e (C)** against CPB.  Superposition of **4a** (beige), **14a** (cyan) and **14e** (pink). The yellow surface represents CYS26 and dark blue is HIS164.
